# Mayahuelin, a Type I Ribosome Inactivating Protein: Characterization, Evolution, and Utilization in Phylogenetic Analyses of *Agave*

**DOI:** 10.3389/fpls.2020.00573

**Published:** 2020-05-27

**Authors:** Fernando Lledías, Jesús Gutiérrez, Aída Martínez-Hernández, Abisaí García-Mendoza, Eric Sosa, Felipe Hernández-Bermúdez, Tzvetanka D. Dinkova, Sandi Reyes, Gladys I. Cassab, Jorge Nieto-Sotelo

**Affiliations:** ^1^Departamento de Biología Molecular de Plantas, Instituto de Biotecnología, Universidad Nacional Autónoma de México, Cuernavaca, Mexico; ^2^Jardín Botánico, Instituto de Biología, Universidad Nacional Autónoma de México, Mexico City, Mexico; ^3^Colegio de Postgraduados, Campus Campeche, Campeche, Mexico; ^4^Departamento de Bioquímica, Facultad de Química, Universidad Nacional Autónoma de México, Mexico City, Mexico

**Keywords:** RIP (ribosome inactivating protein), active site substitution, plant domestication, protein translation, disjoint distributions, agave evolution

## Abstract

Agaves resist extreme heat and drought. In *A. tequilana* var. *azul*, the central spike of the rosette -containing the shoot apical meristem and folded leaves in early stages of development- is remarkably heat tolerant. We found that the most abundant protein in this organ is a 27 kDa protein. This protein was named mayahuelin to honor Mayáhuel, the agave goddess in the Aztec pantheon. LC-MS/MS analyses identified mayahuelin as a type I RIP (**R**ibosome **I**nactivating **P**rotein). In addition to the spike, mayahuelin was expressed in the peduncle and in seeds, whereas in mature leaves, anthers, filaments, pistils, and tepals was absent. Anti-mayahuelin antibody raised against the *A. tequilana* var. *azul* protein revealed strong signals in spike leaves of *A. angustifolia*, *A. bracteosa*, *A. rhodacantha*, and *A. vilmoriniana*, and moderate signals in *A. isthmensis*, *A. kerchovei*, *A. striata* ssp. *falcata*, and *A. titanota*, indicating conservation at the protein level throughout the *Agave* genus. As in charybdin, a type I RIP characterized in *Drimia maritima*, mayahuelin from *A. tequilana* var. *azul* contains a natural aa substitution (Y76D) in one out of four aa comprising the active site. The RIP gene family in *A. tequilana* var. *azul* consists of at least 12 genes and *Mayahuelin* is the only member encoding active site substitutions. Unlike canonical plant RIPs, expression of *Mayahuelin* gene in *S. cerevisiae* did not compromise growth. The inhibitory activity of the purified protein on a wheat germ *in vitro* translation system was moderate. *Mayahuelin* orthologs from other *Agave* species displayed one of six alleles at Y76: (Y/Y, D/D, S/S, Y/D, Y/S, D/S) and proved to be useful markers for phylogenetic analysis. Homozygous alleles were more frequent in wild accessions whereas heterozygous alleles were more frequent in cultivars. *Mayahuelin* sequences from different wild populations of *A. angustifolia* and *A. rhodacanth*a allowed the identification of accessions closely related to *azul, manso, sigüín, mano larga, and bermejo* varieties of *A. tequilana* and var. *espadín* of *A. angustifolia*. Four *A. rhodacantha* accessions and *A. angustifolia* var. *espadín* were closer relatives of *A. tequilana* var. *azul* than *A. angustifolia* wild accessions or other *A. tequilana* varieties.

## Introduction

The *Agave* genus is a member of the Agavoideae subfamily within the Asparagaceae family of plants (The Angiosperm Phylogeny Group, 2009; Chase et al., 2009). The natural distribution of *Agave* encompasses the United States, Mexico, Central America, the Caribbean islands, and South America as far south as Paraguay (García-Mendoza, 1998). The *Agave* genus contains approximately 206 species; Mexico has the highest diversity of species (159, of which 119 are endemic) and it is considered its center of origin (Gentry, 1982; García-Mendoza, 1998; García-Mendoza and Chávez-Rendón, 2013). Most species in the genus are adapted to and play important ecological roles as part of dry ecosystems or arid microenvironments within mesic habitats. *Agave* species also are a food source for bats of the *Leptonycteris* genus that migrate long distances in Mexico and the Sonoran desert (Howell and Roth, 1981; Rojas-Martínez et al., 1999). The cultural importance of agaves in Mexico and the United States Southwest is enormous since pre-historical times to the present. More than 70 known traditional uses are documented for species in the genus (Castetter et al., 1938; Nobel, 1988; García-Mendoza, 1998). In addition, agaves show a great potential as bioenergy crops and as sources of bioactive compounds with anticancer, antioxidant, antimicrobial, antifungal, pre-biotic, and anti-inflammatory properties (Barreto et al., 2010; Escamilla-Treviño, 2011; Simpson et al., 2011; Santos-Zea et al., 2012; Hernández-Valdepeña et al., 2016).

The morphological and physiological adaptations of agaves to high temperature and aridity include succulency of leaves and stems, long and narrow leaves, rosettes sitting near the soil that facilitate nocturnal water collection from dew that is funneled to the base of the plant, shallow roots, thick cuticles, low stomatal densities, and CAM metabolism (Nobel, 1988; Martorell and Ezcurra, 2007; Luján et al., 2009).

In *A. tequilana* var. *azul* the structure with the highest heat resistance is the spike (Luján et al., 2009) which is composed by several folded leaves, located at the center of the rosette, that surround and protect the shoot apical meristem. Heat resistance in the spike is mostly due to its higher levels of heat shock proteins (HSP), higher stomatal density, and greater capacity for leaf cooling relative to more mature sectors of the rosette (Luján et al., 2009). During the progress of the previous study, we detected a 27 kDa protein as the most abundant protein in the spike leaves; we further studied it suspecting to be an HSP. We named this protein mayahuelin after *Mayáhuel*, the agave goddess, a prominent member of the Mesoamerican pantheon, and venerated by ancient náhuatl-speaking cultures (i.e. aztecs, mexicas, etc.) (Tena Martínez, 2002). Amino acid and nucleotide sequence analyses revealed that mayahuelin is a Type I ribosome inactivating protein (RIP) and represents the first RIP described within the subfamily Agavoideae.

Ribosome inactivating proteins are a family of cytotoxic polypeptides with the capacity to bind ribosome large subunits. This interaction causes the irreversible blockage of protein synthesis (De Virgilio et al., 2010). RIPs are found in bacteria, fungi and especially in plants, where they have been described in 22 families representing 14 angiosperm orders (Puri et al., 2012; Di Maro et al., 2014). Most RIPs have a N-glicosidase activity [EC. 3.2.2.22] that removes an adenine residue from the highly conserved rRNA structure known as “Sarcin-Ricin Loop” (SRL). This structure is a central element for the interaction between the ribosome and the Elongation Factor II (EFII). SRL takes its name from ricin, a RIP from *Ricinus communis* seeds that depurinates (A4324) rat 28S rRNA, and from sarcin, a RIP from *Aspergillus giganteus* that breaks the phosphodiester bond between the G4325-A4326 residues of the 28S rRNA (Szewcsak and Moore, 1995; Spackova and Sponer, 2006). Despite SRL structural conservation, RIP specificity for ribosomes shows clear differences (May et al., 2013) while ricin severely damages mammalian and yeast ribosomes, its effects on plants are minimum and null for *E. coli*. In contrast, the “Pokeweed Antiviral Protein” or PAP (a type I RIP from *Phytolacca americana*) equally depurinates plant, bacterial, and animal ribosomes (Peumans et al., 2001). Apart from rRNA, many RIPs depurinate DNA, adenine polynucleotides, and different viral RNAs. Because of this multi-substrate activity, RIPs are also known as “Polynucleotide-Adenosine Glicosidases” (Barbieri et al., 1997).

Ribosome inactivating proteins are classified as type I, formed by one chain named A (MW 30 kDa), and type II, heterodimers between an A chain (type I-like) and a B chain with lectin properties. Both chains have a MW from 56 to 65 kDa (Stirpe and Batelli, 2006). A 60 kDa type III RIPs (or pro-RIPs) with a type I N-terminus, and a C-end with unknown function has been described as an inactive precursor that requires processing to obtain a functional RIP (Puri et al., 2012). Type II RIPs are highly toxic on account of the B chain, that promotes entry of the A chain into the cell (Stirpe, 2013). In some plant species RIPs are widely expressed in different tissues (e.g. saporin from *Saponaria oficinalis* is found in leaves, roots and seeds), while in others show tissue-specific location (e.g. ricin from *R. communis* found in seeds only).

Ribosome inactivating protein first enzymatic mechanisms were elucidated in ricin A chain, where the catalytic site residues responsible for SRL depurination were identified as Y80, Y123, E177, and R180 (Kim and Robertus, 1992). Catalytic site amino acids and their tertiary structure are highly conserved in at least 10 published RIP crystal structures (Peumans et al., 2001). Individual catalytic site amino acid substitutions have different impact on enzymatic activity of RIPs. The R180H substitution in ricin rearranges the active site and decreases activity 500-fold (Day et al., 1996) whereas substitutions in active site tyrosine residues, Y80S or Y123S, in charge of adenine-substrate stabilization, decrease depurination activity by 160- and 70-fold, respectively, (Ready et al., 1991; Kim and Robertus, 1992).

Ribosome inactivating protein expression increases under different stress conditions, during senescence, and in response to microbial and viral infections (Stirpe and Batelli, 2006). Under osmotic and heat stress, the translation inhibitory activity and DNA deadenylation activities of RIPs increase in *Hura crepitans* and *Phytolacca americana* (Stirpe et al., 1996). Moreover, RIP overexpression increases drought and salt tolerance in rice, this gain attributed to the up-regulation of stress genes through unknown mechanisms (Jiang et al., 2012). Accordingly, exogenous administration of purified RIP from *P. americana* on tobacco leaves protects them from tobacco mosaic virus infection and increases the levels of antioxidant enzymes (Zhu et al., 2016).

Several *Agave* molecular phylogenies have been proposed based on nucleotide sequences derived from *rbc*L, *trn*L+*trn*L-*trn*F, *ndh*F, and *ITS* markers (Bogler and Simpson, 1996; Bogler et al., 2006; Good-Avila et al., 2006; Heyduk et al., 2016; Huang et al., 2018) and have been very useful to resolve different genera within Agavoideae (*Agave*, *Beschorneria*, *Furcraea*, *Hesperaloe*, *Hesperoyucca*, *Manfreda*, *Polianthes, Prochnyanthes*, and *Yucca*). However, these markers offer very poor intrageneric resolution. In contrast, AFLP, SSR, and SSAP markers offer very high genetic resolutions, allowing population genetic analyses in different *Agave* species (Gil-Vega et al., 2006; Bousios et al., 2007; Trejo et al., 2018; Rivera-Lugo et al., 2018). Because false positive and false negative data can be obtained using fragment length as an estimate for genetic identity between individuals, especially when dealing with genetically distant species, methods alternative to AFLP, SSR, and SSAP have been proposed to derive molecular phylogenies based on nucleotide sequence, such as transcriptomics, RNA/DNA hybrid enrichment, and phylogenomics, all of which are based on next-generation sequencing (Lemmon and Lemmon, 2013; Huang et al., 2018; Zhang et al., 2018; Fernández et al., 2018). However, they are very costly, time-consuming, and not very practical when large numbers of samples are studied. Moreover, the large number of genetic markers available with these methods does not warrant phylogenetic resolution, as shown by a recent study of CAM (Crassulacean acid metabolism) evolution in Agavoideae that used 272 CAM-related genes to derive a phylogeny of 60 Agavoideae species (Heyduk et al., 2016) giving good resolution at the intergeneric level, but poor resolution at the intrageneric level, displaying hard polytomies in *Agave sensu lato*. Thus, nuclear markers that allow sufficient genetic resolution at the species level are still needed for plant phylogenetic analysis. *A. tequilana* var. *azul*, the exclusive cultivar approved for tequila production according to Mexican law, is cultivated asexually to maintain its varietal qualities; as a result its populations have extremely low genetic diversity (Gil-Vega et al., 2001). Hence, it is critical to identify the wild populations that originated this variety and other clonally propagated cultivars used for. tequila and mescal production. This knowledge is fundamental for both their conservation and improvement as they represent potential sources of genetic variation. Phylogenetic methods are very powerful tools to achieve these goals.

Here, we present results on the isolation, characterization, expression, and evolution of mayahuelin and its use as a phylogenetic marker within the *Agave* genus. We report the expression analyses of mayahuelin in different plant organs. Interestingly, a highly conserved tyrosine (Y76) in the active site of all RIPs (corresponding to Y80 in ricin) was naturally mutated to aspartate or serine in mayahuelin from *A. tequilana* var. *azul* and in other species within the *Agave* genus. Mayahuelin from *A. tequilana* var. *azul* containing the Y76D substitution was not toxic *in vivo* when expressed in yeast and only moderately toxic *in vitro*. Mayahuelin was immunodetected in the spike of several species of both the Littaea and Agave subgenera, indicating conservation at the protein level throughout the genus. Phylogenetic analyses using *Mayahuelin* ortholog sequences identified accessions of *A. rhodacantha* and *A. angustifolia* as close relatives of five *A. tequilana* and one *A. angustifolia* cultivars. Several accessions of *A. rhodacantha* and *A. angustifolia* intermixed in different *Agave* clades and were more genetically distant from these cultivars. We discuss the implications of the Y76 substitutions in terms of *Agave* as a natural resource and in domesticated plants.

## Materials and Methods

### Plant Materials

Plant materials utilized in this work came from different sources: the National Collection of Agavaceae and Nolinaceae from Jardín Botánico, Instituto de Biología, Universidad Nacional Autónoma de México, in Mexico City; the agave collection of Jardín Botánico, Casa Sauza, in Tequila, Jalisco, Mexico, and from recent field trips made for this work (for a detailed list of plants studied and their sources see Supplementary Table S1 and Supplementary Figure S1).

### Mayahuelin Native Protein Purification

Native mayahuelin was obtained from fresh *A. tequilana* var. *azul* spike leaves cut with scissors and pulverized with a mortar and pestle in liquid nitrogen. 1 mL of frozen tissue powder was transferred to 2 mL tubes and 1 mL extraction buffer (200 mM Tris pH 7.2, 20 mM NaCl, 0.5% (v/v) β-mercapthoethanol, 2 mM EDTA pH 8.0, and 10X Complete protease inhibitor cocktail [Roche]). Tissue was resuspended with a stainless steel spatula, while thawing for 30 s. Each tube was perforated at the bottom with a syringe needle and placed atop 15 mL conic tubes and centrifuged (6 000 × *g* for 10 min, 6°C) to recover the liquid phase, taking the advantage that *A. tequilana* natural fibers worked as a filter. Supernatants were recovered and two volumes of cold acetone were added. After 30 min on ice, tubes were centrifuged (14,000 × *g* for 10 min, 25°C) and supernatants were discarded. Pellets were air-dried (30 min) and resuspended in 200 mM Tris pH 8.8, 1% glycerol. Tubes were centrifuged again. Supernatants were recovered for their separation on native gels (see Supplementary Information).

### Mass Spectrometry

A published protocol for preparation of total protein extracts from *Agave*, separation by electrophoresis, and mass spectrometric analysis was followed (Lledías et al., 2017a, b). Samples recovered from “Little blue tank” (see Supplemental Information) traps were precipitated with methanol/chloroform, resuspended in 1X Laemmli sample buffer and directly loaded onto a stacking gel of a 12% polyacrylamide/SDS. The resulting band was excised for analysis. In-gel samples were chemically modified prior to mass spectrometry analysis. After reduction (dithiothreitol) and alkylation (iodoacetamide), samples were digested in gel with sequencing grade modified trypsin (Promega; Madison, WI, United States) in a solution of 50 mM ammonium bicarbonate pH 8.2 for 18 h at 37°C. Resultant peptides were desalted with Zip Tip C18 (Millipore; Billerica, MA, United States) and applied to a LC-MS system (Liquid Chromatography-Mass Spectrometry) composed by an EASY-nLC II nanoflow pump (Thermo Fisher Scientific; San Jose, CA, United States) coupled to a LTQ-Orbitrap Velos (Thermo Fisher Scientific; San Jose, CA, United States) mass spectrometer with a nano-electrospray ionization (ESI) source. The mass spectrometer was calibrated with a Calmix solution containing N-butylamine, caffeine, Met-Arg-Phe-Ala (MRFA) peptide, and Ultramark 1621. This solution was used to calibrate the LTQ Velos module with ion trap (IT) and Orbitrap FT (Fourier transform) mass detector on positive ionization ESI mode. N-butylamine (73.14 Da) was included to extend mass calibration to values less than *m*/*z*. Once calibrated, molecular mass accuracy at less than 5 ppm can be obtained. For LC, a 5%–85% gradient of solution B (water/acetonitrile, 0.1% formic acid) and solvent A (0.1% formic acid in water) was used for 160 min through a home-made capillary column (10 cm in length, ID 0.75 μm) made of TSP standard FS tubing with OD 363 μm (part no. TSP-075375BGB, Analytik, United States) packed with a C18-reversed phase silica gel (Jupiter 4 μm Proteo 90 Å, Phenomenex; Torrance, CA, United States) with a flux of 10 μL/min. For peptide fragmentation, Collision-Induced Dissociation (CID) and High-energy Collision Dissociation (HCD) methods were used with a resolution power (RP = m/FWHM) of 15,000 and selecting only 2+, 3+ and 4+ charged ions. A full scan of ions was performed with the Orbitrap analyser with a resolution power (RP = m/FWHM) of 60,000. For data acquisition, the positive ion mode was set. Capture and performance of fragmentation data were done according to the total ion scanning and predetermined charge with the following parameters: 2.0 (m/z) isolation width; collision energy, 35 arbitrary units; activation Q, 0.250; activation time, 10 ms; maximum injection time, 10 ms per micro-scanning. The automatic capture of data was done by ion dynamic exclusion: (i) exclusion list of 300 ions; (ii) pre-exclusion time of 30 s; and (iii) exclusion time of 70 s. Sequences obtained by electrospray LC-MS/MS were searched in.raw format with the Proteome Discoverer 1.4.1.14 (Thermo Fisher Scientific; San Jose; CA, United States) and the search engine Sequest HT. Since proteomic data in Agavoideae is lacking, we searched an EST library database from *A. tequilana* var. *azul* (Martínez-Hernández et al., 2010; Simpson et al., 2011). A minimal FDR (false discovery rate) of 0.01 and maximal FDR of 0.05, in addition to a decoy database, were used in the *Percolator* program. The maximum tolerance for molecular mass differences between theoretical and experimental values (precursor mass tolerance) was 20 ppm; tolerance for fragments obtained afer dissociation of precursor ion (fragment mass tolerance) was 0.6 Da. For automatic search mode, modification constants such as carbamido-methylation of cysteines (C) and variables such as methionine oxidation (M), asparagine (N) and glutamine (Q) deaminations were established.

N-terminal sequencing of mayahuelin, isolated by the native protein purification protocol described above, was accomplished by Edman degradation using an LF 3000 (Beckman Instruments, Irvine, CA, United States) automated protein sequencer coupled to a Beckman GoldHPLC system.

### Other Methods for Protein Biochemical Analysis

Detailed protocols for native gel electrophoresis, native protein electroelution, polyclonal antibody production, SDS-PAGE analysis, and immunoblot analysis of mayahuelin are described in Supplementary Information.

### Evaluation of the Effects of Mayahuelin on Protein Translation *in vitro*

The inhibitory effect of mayahuelin on protein translation was tested using luciferase as a reporter on a cell-free wheat germ protein synthesis system (cat. L4380, Promega). Mayahuelin was tested at different nM concentrations and, as a positive control, saporin [13.3 nM] was tested (cat. S9896, Sigma-Aldrich). Samples were preincubated at 25°C for 30 min with RNAsin, an RNAase inhibitor (cat. N2111, Promega). After preincubation, 50 ng of luciferase coding RNA were added and kept for an extra 1.5 h at 25°C. The reaction was stopped with 10 μL of 1X passive lysis buffer (cat. E1941, Promega). Luciferase reactant (50 μL) (cat. E1483, Promega) was added to 10 μL aliquots from each reaction, in triplicate. As a negative control, one reaction without protein and mRNA was included. Samples were analyzed on a Varioskan Lux 3020-176 luminometer (Thermo Fisher Scientific). Data was analyzed by one-way ANOVA using *GraphPad Prism* v6 software. To determine mayahuelin IC_50_, data was adjusted to a non-linear dose-response curve using the logistic fitting with four parameters function (Origin v9.6 software).

### Mayahuelin Homology Modeling

Mayahuelin amino acid sequence from *A. tequilana* var. *azul* was used to fetch protein sequences with the *BLASTP* program^1^ restricting the search only to proteins with known X-ray crystalographic structures resolved at 2.5°A or lower and accepting only outputs with an identity larger than 30 and >90% coverage. Charybdin from *Drimia maritima* [sea onion, Asparagaceae, subfamily Scilloideae] (Touloupakis et al., 2006) obtained the best hit with 37% identity and 92% coverage, relative to mayahuelin. Charybdin structure was retrieved as a.pdb file from the Protein Data Bank^2^ and additional lateral chains, ions, and ligands were removed to obtain the basic frame of the protein. Protein alignment between the amino acid sequences of mayahuelin and charybdin was performed with *T-COFFEE* v11.0 and hand edited before conversion to.pir format. Both.pir and.pdb files were uploaded to the *Modeller* v9.19 program generating 10,000 mayahuelin models. Only the top three models – those having the lowest DOPE (Discrete Optimized Protein Energy) scores – were further considered for analysis (Shen and Sali, 2006). The selected models were evaluated with *ERRAT*^3^, a tool designed to identify protein regions on need of refinement. The model with the highest score was subject to refinement in regions with >99% rejection. Using *Modeller*, 1,000 new refined models were created by repeating a new cycle of evaluations with DOPE and ERRAT to identify the model with the best score. The final model was evaluated on a Ramachandran graph using *Molprobity*^4^.

### Other Molecular Biology Methods Used

Protocols for the cloning and expression of *Mayahuelin* gene in *S. cerevisiae*, for the estimation of *Mayahuelin* transcript levels, and for the amplification of *Mayahuelin* genes by RT-PCR and direct PCR are described in Supplementary Information.

### Mayahuelin Orthology Tests

Protein-coding nucleotide sequences of *Mayahuelin* candidates were aligned together with the 12 RIP family members of *Agave tequilana* var. *azul* (Supplementary Figures S2, S3, S11, S12, and Supplementary Table S4). We used *TranslatorX*, a multiple-alignment method based on the corresponding aa alignments encoded by such sequences. *TranslatorX* first performs the aa aligment and from the output optimizes the aligment of the nucleotide sequences (Abascal et al., 2010). As an additional parameter, the algorithm *MUSCLE* was used (Edgar, 2004). The resulting multiple alignments were analyzed by both Maximun-likelihood (ML) and Bayesian inference (BI) methods.

The program *PhyML* v3.0 (Guindon et al., 2010)^5^ was used to perform ML. To obtain the best nucleotide substitution reconstruction the *SMS* algorithm was selected (Lefort et al., 2017) and implemented in *PhyML*. Default values were used for construction of the starting tree (BIONJ option) and for tree improvement (NNI, Nearest-neighbor interchange option). Support values were obtained by bootstrapping with 1,000 pseudoreplicates.

BI analyses were performed with MrBayes v3.2 (Ronquist et al., 2012). To analyze the sequences based on codons, instead of single nucleotides, the *GTR* (General Time Reversible) nucleotide substitution refinement was selected with the *codon* option. Other parameters were used under default values and considering aC3095_122 RIP sequence as outgroup. Two independent and simultaneous Markov chain Monte Carlo simulations (MCMC) were run using six hot chains and two cold chains; random starting trees, sampling of refinement parameters, posterior probabilites every 500 generations, and discarding 25% of initial generations (burn-in) were implemented. A total of 1e+06 generations were run. Convergence of the two chains was determined by examining the average standard desviation of splites frequencies in MrBayes and by calculating the efective sample size (ESS) with the program *Tracer* v1.7.1 (Rambaut et al., 2018). An standard deviation <0.01 and a total ESS >200 were used as criteria to establish convergence in the stationary phase.

Using either ML or BI methods, only candidate sequences that clustered with clone aC630_3 (*Mayahuelin*) were accepted as orthologs for phylogenetic analysis.

### Phylogenetic Analyses

A panel of 34 taxa within the Littaea and Agave subgenera was assembled (see Supplementary Tables S1, S6) with a focus on *A. tequilana* and other members of the Rigidae group (*A.angustifolia*, *A. rhodacantha*, and *A. aktites*) in addition to members of the Hiemiflorae (*A. isthmensis*), Americanae (*A. americana*), Parryanae (*A. guadalajarana* and *A. parryi*), and Marmoratae (*A. zebra*) groups within the Agave subgenus as well as the Littaea subgenus represented by Choripetalae (*A. guiengola*), Amolae (*A. vilmoriniana*) and Marginatae (*A. horrida*) groups. *Beschorneria calcicola* was chosen as an outgroup. *B. calcicola* is a member of Agavoideae consistently found in separate clades that stem from more basal nodes relative to the *Agave* genus in all published molecular phylogenies (Bogler and Simpson, 1996; Bogler et al., 2006; Good-Avila et al., 2006; Heyduk et al., 2016). The selection considered both cultivated and wild individuals: five cultivars of *A. tequilana* (*azul*, *manso*, *sigüín*, *mano larga*, and *bermejo*), three cultivars of *A. angustifolia* (*espadín*, *Huajuapan*, and *Ahuacuotzingo*), four cultivars of *A. rhodacantha* (*ixtlero amarillo*, *Ejutla*, *UNAM*, and *Nayarit*), seven wild accessions of *A. angustifolia* (from Sonora, Sinaloa, Jalisco, Guerrero, and Oaxaca), five wild accessions of *A. rhodacantha* (from Sonora, Sinaloa, Jalisco, and Oaxaca), nine additional *Agave* species with or without current/pre-historical evidence of utilization by humans (*A. americana*, *A. aktites*, *A. guadalajarana*, *A. guiengola*, *A. horrida*, *A. isthmensis*, *A. parryi*, *A. vilmoriniana*, and *A. zebra*), and *B. calcicola*.

*Mayahuelin* F2-R2 or F6-R6 specific primer pairs (Supplementary Table S3 and Supplementary Figure S8), complementary to gene sequences encoding the N- and C- terminal ends of mature mayahuelin, were used to amplify *Mayahuelin* orthologs by either RT-PCR or direct genomic PCR. None of the amplified genomic sequences contained introns. *Mayahuelin* paralog sequences were excluded from the analyses (see Supplementary Figures S4, S12 section for orthology tests). Validated sequences were aligned with the *TranslatorX* and *MUSCLE* programs (Edgar, 2004; Abascal et al., 2010). After trimming, the length of the alignment spanned 630 bp yielding 104 variable sites with a proportion of 0.165 (Table 1). The consensus tree for Maximum-likelihood was inferred with *PhyML* 3.0 and by bootstrapping with 1,000 pseudoreplicates, as described above. For BI analyses, a consensus tree was derived with MrBayes program. The *GTR* reconstruction was selected under the *codon* option using *B. calcicola* sequence as an outgroup. A total of 1.2e+06 generations of MCMC simulations were run. Other parameters and convergence measurements were used as described above.

**TABLE 1:** A summary of the sequence alignment of 34 Agavoideae taxa.

No. of taxa	34
Alignment length (bp)	741
Alignment_length after trimming (bp)	630
Undetermined characters	216
Missing percent	1.008
Number of variable sites	104
Proportion of variable sites	0.165
Singleton sites (bp)	62
Parsimony informative sites	42
Proportion of parsimony informative sites	0.067
AT content	0.47
GC content	0.53

*Data were obtained with the AMAS software (Borowiec, 2016).*

## Results

### Mayahuelin Is Abundant in Spike Leaves and Seeds

**FIGURE 1:**
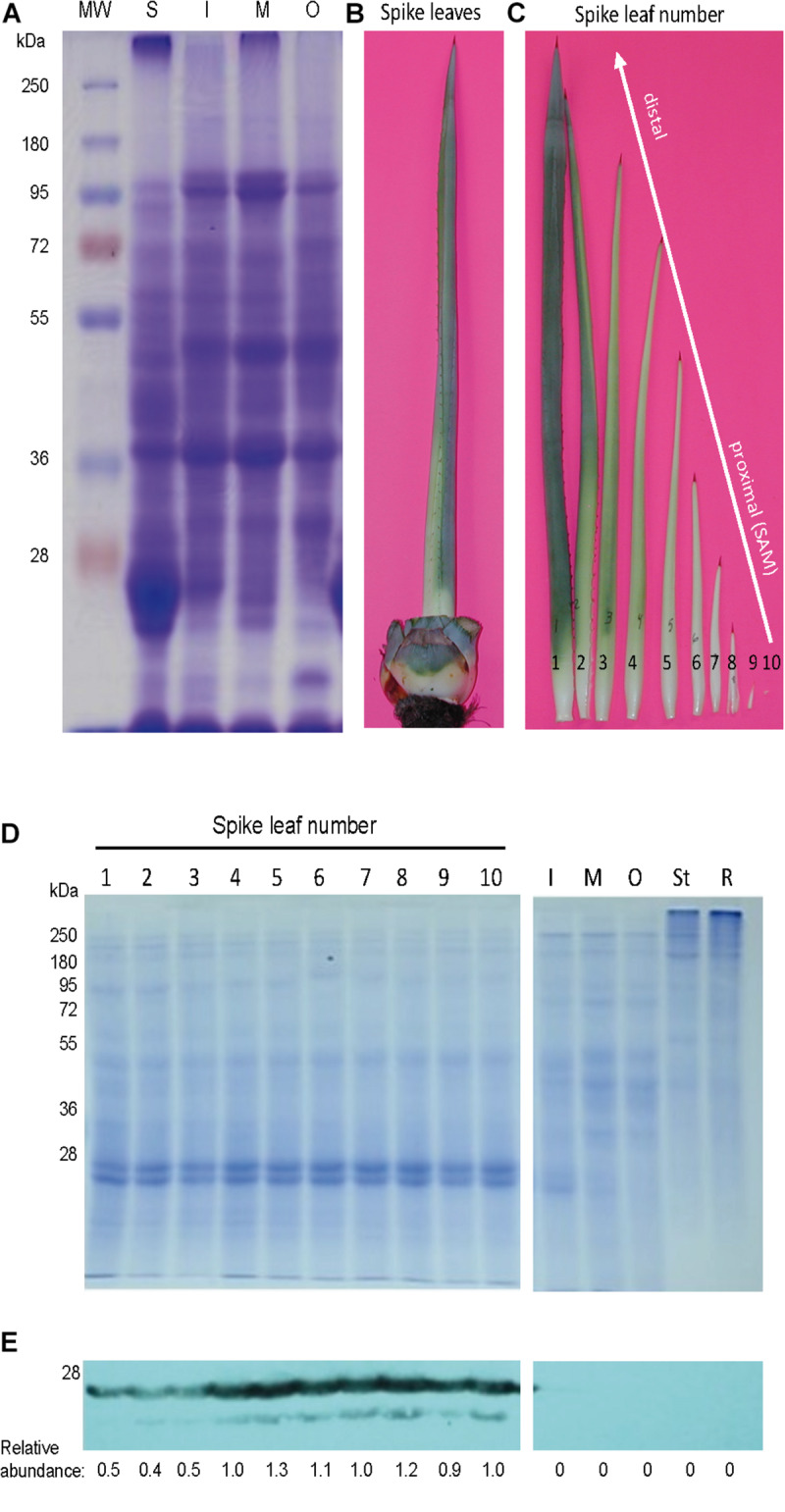
In the rosette of *A. tequilana* var. *azul*, mayahuelin is exclusively expressed in spike leaves. **(A)** SDS/PAGE profile of total proteins (20 μg) in leaves from the spike (S) and from the internal (I), medium (M) and outer (O) regions of an *A. tequilana* var. *azul* rosette. The spike **(B)** consists of several folded leaves in different stages of development **(C)**. To estimate the levels of mayahuelin, total protein extracts (5 μg) were individually analyzed by SDS/PAGE **(D)** and by immunobloting using an anti-mayahuelin antibody **(E)**. Mayahuelin levels are high in spike leaves (**E,** left image). In leaves from the internal (I) medium (M), and outer (O) regions, or in tissues from the stem (St) and roots (R), mayahuelin was absent (**E**, right image). Protein levels shown in **(E)** panel are relative to spike leaf number 10.

**FIGURE 2:**
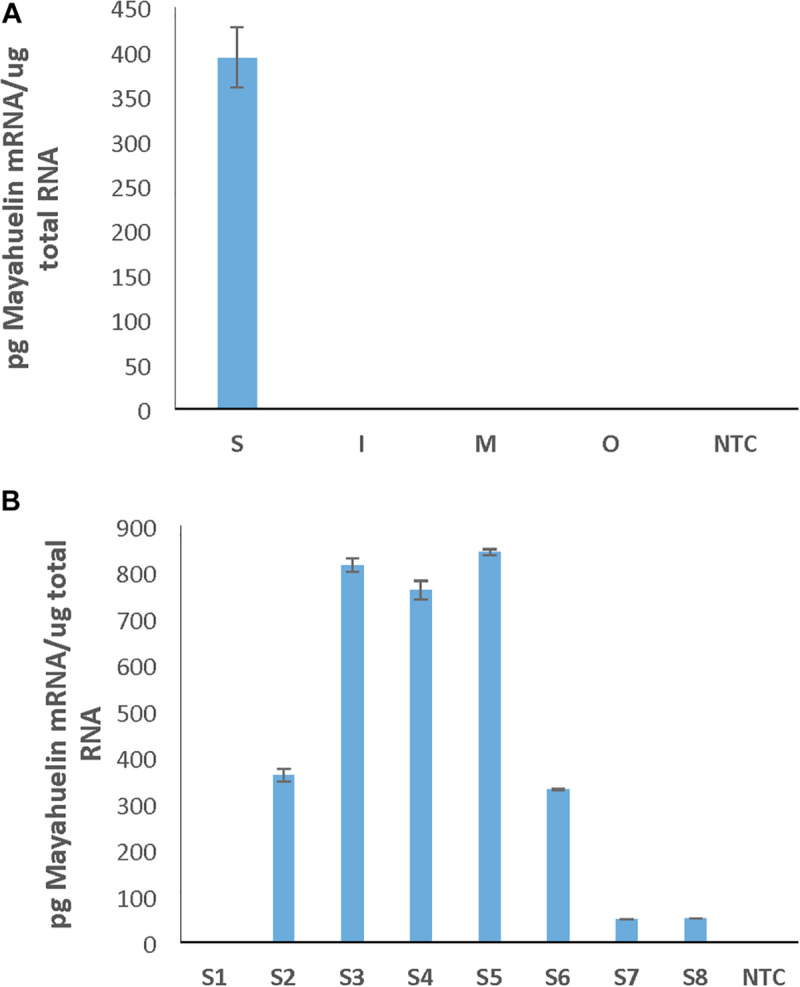
Mayahuelin transcripts accumulate only in spike leaves of *A. tequilana* var. *azul*. Quantitative PCR assays were performed using total RNA isolated from leaves from different rosette sectors (**S**pike, **I**nternal, **M**edium, and **O**uter) **(A)** or from individual leaves of the spike (leaves S1–S8 represent a developmental leaf gradient, where leaf S1 is the most mature one and S8 the youngest one and the leaf closest to the SAM) **(B)**. NTC, no template control reaction. The spike of the *A. tequilana* specimen used in this study contained only 8 leaves and came from a different specimen to the one used in experiment on Figure 1.

**FIGURE 3:**
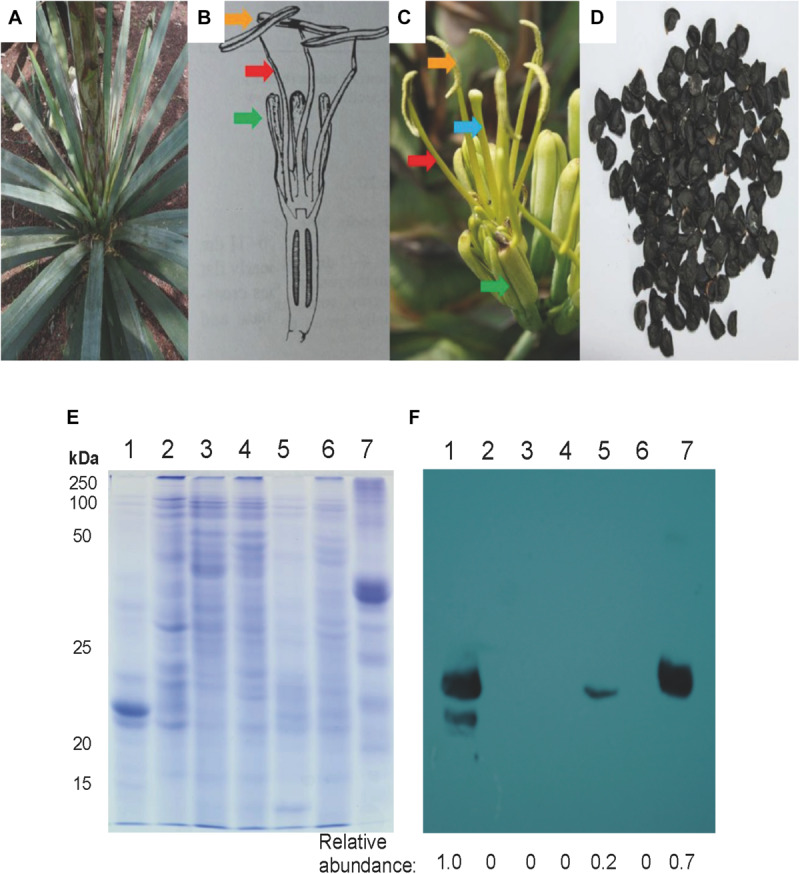
Mayahuelin levels in *A. tequilana* var. *azul* mature/reproductive plant organs. Upper portion: Images of a whole plant **(A)**, a schematic representation and image of a flower **(B,C)**, and seeds **(D)** from *A. tequilana* var. *azul* plants used for analyses. In **(B,C)** green, red, blue, and orange arrows point tepals, filaments, pistil, and anthers, respectively. Lower portion: SDS/PAGE **(E)** and immuno-blot **(F)** analyses of total protein preparations from spike leaves (lane 1), anthers (lane 2), filaments (lane 3), pistils (lane 4), flowering spike (lane 5), tepals (lane 6) and seeds (lane 7). Protein levels shown in **(F)** panel are relative to spike leaves sample.

Agaves form a spirally shaped rosette; at its center, a spike is visible (a group of folded leaves that surround and protect the shoot apical meristem) and several unfolded and more mature leaves in the periphery. During the progress of development of an *ad hoc* method for total protein extraction from *A. tequilana* var. *azul* leaves (Lledías et al., 2017a, b), we noticed a highly accumulated 27 kDa molecular mass protein present exclusively in the spike and we named this protein mayahuelin (Figure 1A). This structure is formed by a variable number of leaves (approximately 8 to 15, Figures 1B,C). Protein from each spike leaf was individually analyzed to estimate mayahuelin content. A strong signal was detected in leaves 10 to 4 (in a proximal to distal order, relative to the shoot apical meristem [SAM]) and lower levels in leaves 3 to 1 (Figures 1C–E). In samples from internal (I), middle (M), and outer (O) rosette sectors, as well as in stem (St) and roots (R), mayahuelin was absent (Figures 1D,E, right panels). Qualitative estimation of *Mayahuelin* RNA accumulation by quantitative RT-PCR indicated that *Mayahuelin* transcripts were expressed in spike leaves only and that their levels were near absent in leaf 1, reaching a peak in leaves 3 to 5, and levels decreasing in leaves 7 and 8 (Figure 2). Analyzed tissues from a mature *A. tequilana* var. *azul* plant (Figure 3A) undergoing sexual reproduction showed a high mayahuelin protein content (equivalent to spike leaves) in seeds (Figure 3D–F, lane 7). A faint signal was also observed in the floral peduncle (Figures 3E,F, lane 5). Mayahuelin was absent in floral structures (Figures 3B,C,E,F, lanes 2, 3, 4, and 6).

### Mayahuelin Purification

**FIGURE 4:**
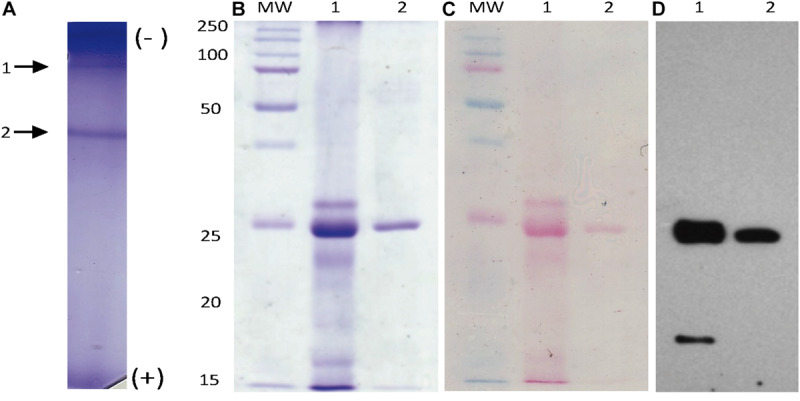
Mayahuelin native-protein purification. Proteins from spike supernatants were separated by native PAGE **(A)**. After Coomassie blue-staining, two main bands were observed (arrows 1 and 2 in **A**). Band 2 (mayahuelin) was electroeluted, denatured, and analyzed by SDS/PAGE (**B**, lane 2). Mayahuelin was the major band (27 kDa) detected in a total protein preparation from spike leaves of *A. tequil*ana var. *azul* (**B**, lane 1). A total protein extract from spike leaves of *A. tequil*ana var. *azul* and purified mayahuelin were transferred to nitrocellulose filters, stained with Ponceau red (**C**, lanes 1 and 2) and probed with polyclonal immunoadsorbed rabbit-anti-mayahuelin antibodies (**D**, lanes 1 and 2).

Native electrophoresis of solubilized proteins obtained from spike leaves (after acetone precipitation) revealed a pattern of two major bands (Figure 4A). Band 2, recovered by native electroelution, methanol/chloroform precipitation, and analyzed by SDS/PAGE, appeared as a unique 27 kDa molecular mass protein (Figure 4B, lane 2) that comigrated with the major band present in a total protein preparation from *A. tequilana* var. *azul* from spike leaves (Figure 4B, lanes 1 and 2). The native protein, cross-linked to the polyacrylamide matrix, was used to obtain polyclonal antibodies for immunoblot analysis. A strong signal detected by the anti-mayahuelin sera was observed from protein band 2 (mayahuelin) obtained by electroelution (Figures 4C,D, lane 2) and from the 27 kDa protein present in total protein extracts made from spike leaves (Figures 4C,D, lane 1).

### Accumulation of Mayahuelin in Spike Leaves Is Conserved in the *Agave* Genus

**FIGURE 5:**
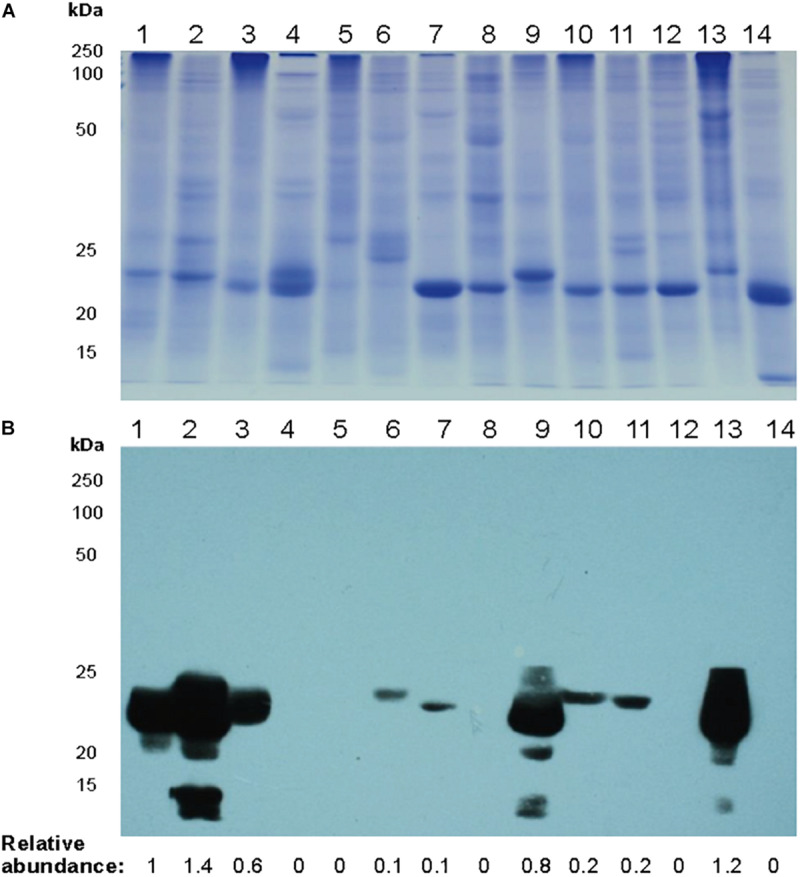
Relative levels of mayahuelin in different *Agave* plant species. **(A)** SDS/PAGE of total protein extracts obtained from spike leaves of *Agave* species. **(B)** Immunoblot analysis of protein extracts [10 μg] obtained from spike leaves of *Agave* species using *A. tequilana* var. *azul* anti-mayahuelin antibody. *A. tequilana* var. *azul* (lane 1); *A. angustifolia* (lane 2); *A. bracteosa* (lane 3); *A. desertii* (lane 4); *A. guiengola* (lane 5); *A. isthmensis* (lane 6); *A. kerchovei* (lane 7); *A. petrophila* (lane 8); *A. rhodacantha* (lane 9); *A. striata* ssp. *falcata* (lane 10); *A. titanota* (lane 11); *A. victoriae-reginae* (lane 12); *A. vilmoriniana* (lane 13); and *A. zebra* (lane 14). Protein levels shown in b panel are relative to A. *tequilana* var. *azul* sample.

We searched for mayahuelin presence in spike leaves total protein extracts from a panel of species of the subgenera Littaea [*A. bracteosa*, *A. desertii*, *A. guiengola*, *A. isthmensis*, *A. kerchovei*, *A. striata sp. falcata, A. titanota*, *A. victoriae-reginae*, and *A. vilmoriniana*] and Agave [*A. angustifolia*, *A. petrophila*, *A. rhodacantha*, and *A. zebra*] (Figure 5). Mayahuelin was detected both in the Littaea and Agave subgenera. In *A. angustifolia*, *A. bracteosa*, *A. rhodacantha*, and *A. vilmoriniana* spike leaves, mayahuelin content was similar or above that in *A. tequilana* var. *azul* (Figure 5B, lanes 1, 2, 3, 9, 13). Low levels of mayahuelin were observed in spike leaves of *A. isthmensis*, *A. kerchovei*, *A. striata sp. falcata*, and *A. titanota* (Figure 5B, lanes 6, 7, 10, and 11). In *A. deserti*, *A. guiengola*, *A. petrophila*, *A. victoriae-reginae*, and *A. zebra* spike leaves (Figure 5B, lanes 4, 5, 8, 12, and 14) mayahuelin levels were below detection even at longer film exposure times.

### Mayahuelin Protein and Gene Sequencing

**FIGURE 6:**
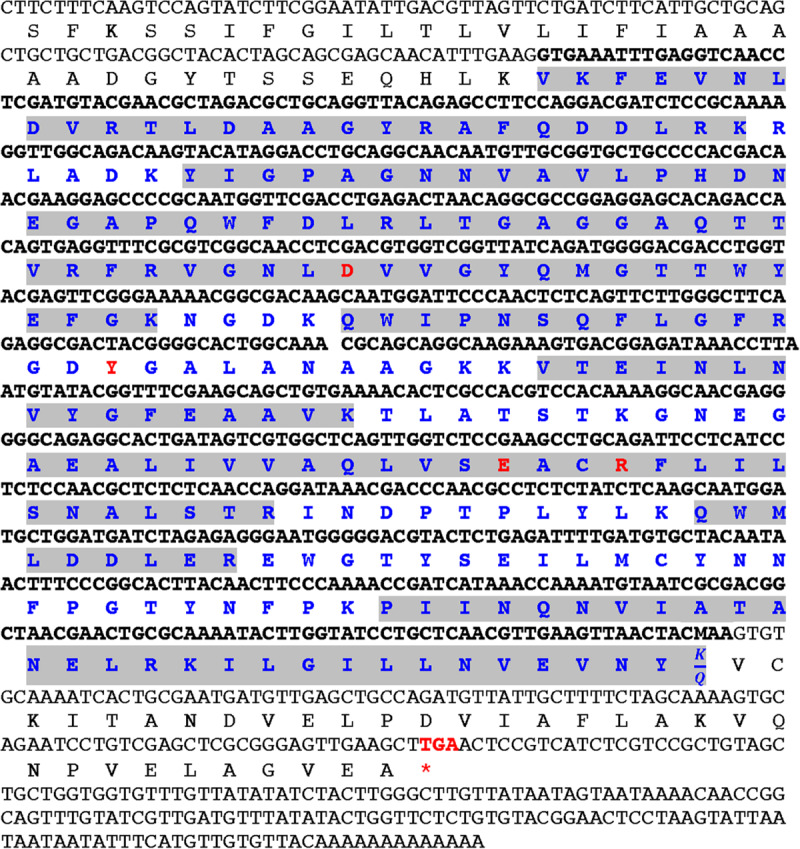
Alignment of mayahuelin nucleotide and amino acid sequences from *A. tequilana* var. *azul*. The nucleotide sequence of clone aC630_3 from an *A. tequilan*a var. *azul* cDNA library is shown in gray or black fonts. Predicted and experimentally determined (LC-MS/MS) amino acid sequences are shown below the nucleotide sequence. Highlighted in gray are the peptide sequences obtained by LC-MS/MS of pure mayahuelin isolated from spike leaves. Red codon (stop) indicates the end of the open reading frame in *Mayahuelin* cDNA. Bold letters in nucleotide and amino acid sequence indicate sequences of mature mayahuelin protein isolated from spike leaves. Red labeled amino acids show the position of the four conserved amino acids in RIP proteins. *Mayahuelin* Genbank accession number is MN913554. Mass spectrometry data was deposited at the Peptide Atlas repository (http://www.peptideatlas.org/repository/) under accession number PASS01536.

To determine the primary aa sequence of mayahuelin, the purified protein was analyzed in two independent biological replicas by mass spectrometry (LC-MS/MS). *De novo* peptide sequencing of mayahuelin fragments (for experiment 2 see Supplementary Table S2, Supplementary Information) from both analyses showed complete identity with cDNA consensus sequence aC630_3 reconstructed from an EST *A. tequilana* var. *azul* library (Martínez-Hernández et al., 2010; Simpson et al., 2011). N-terminal sequencing of mayahuelin was accomplished by Edman degradation. After 15 cycles, the amino acid sequence obtained was VKFEVNLDVRTLXAA. This sequence matched perfectly well with two peptides (# 1 and # 2) sequenced by LC-MS/MS (Supplementary Table S2). One peptide from the first experiment (data not shown) sequenced by LC-MS/MS contained Q at its C-terminus; we assumed that it represented the C-terminus of the protein since it lacked R or K. In the second experiment, this peptide contained K at the C-terminus (peptide # 14 in the second experiment shown in Supplementary Table S2). The analyses of aC630_3 ESTs confirmed the presence of transcripts containing either AAA or CAA codons at this position, explaining the two versions obtained by LC-MS/MS. Therefore, the calculated molecular mass of mayahuelin is 27,251.93, matching very well mayahuelin’s apparent molecular weight calculated by SDS-PAGE. Thus, mayahuelin aa sequence obtained by MS, after the assembly of 14 peptide sequences, covered 73% of the protein (Figure 6 and Supplementary Table S2). The alignment of mayahuelin aa sequence obtained by MS and the sequence predicted by cDNA sequence aC630_3 suggested that mayahuelin was synthetized as a precursor of 310 amino acids in length to which the N- and C- termini were removed (Figure 6). Therefore, mature mayahuelin primary sequence consists of 245 amino acids; the N-terminal end of mayahuelin precursor protein contains a putative signal peptide for extracellular secretion (Supplementary Figures S5A,B). Since cDNA sequence aC630_3 is missing the initiation codon at the 5′ end, mayahuelin precursor must be longer in length (Figure 6). A protein BLAST search at the NCBI database showed that mayahuelin sequence matches to a group of proteins known as Type I RIPs (Ribosome Inactivating Proteins) (Supplementary Figure S6). Protein sequence alignment to known RIPs revealed an amino acid substitution in the active site of mayahuelin: an aspartate replacing a highly conserved tyrosine (Y76D) (Figure 6 and Supplementary Figure S6) as a consequence of a single base change in the tyrosine codon (TAC→GAC). This amino acid change resembles a substitution in charybdin, a 29 kDa Type I RIP from the sea squill plant, (*Drimia maritima*) that shows a valine substitution at the same tyrosine in the active site (Touloupakis et al., 2006; Figure 6 and Supplementary Figure S6). *D. maritima* is the accepted name for its synonym *Charybdis maritima* and it is a member of the Asparagaceae family as *A. tequilana*. The toxicity of charybdin is low in a mouse reticulocyte *in vitro* translation system compared with a canonic RIP like saporin (Touloupakis et al., 2006). A search for *Mayahuelin* related sequences in the *A. tequilana* var. *azul* EST library (Martínez-Hernández et al., 2010; Simpson et al., 2011) uncovered eleven additional cDNA sequences encoding putative RIPs. Analysis of their open reading frames showed that *Mayahuelin* is the only member of the *A. tequilana* var. *azul* RIP family encoding substitutions in the active site of the protein (Supplementary Figure S2, Supplementary Information).

### Mayahuelin Expression Is Harmless for the Growth of *S. cerevisiae* Cells

**FIGURE 7:**
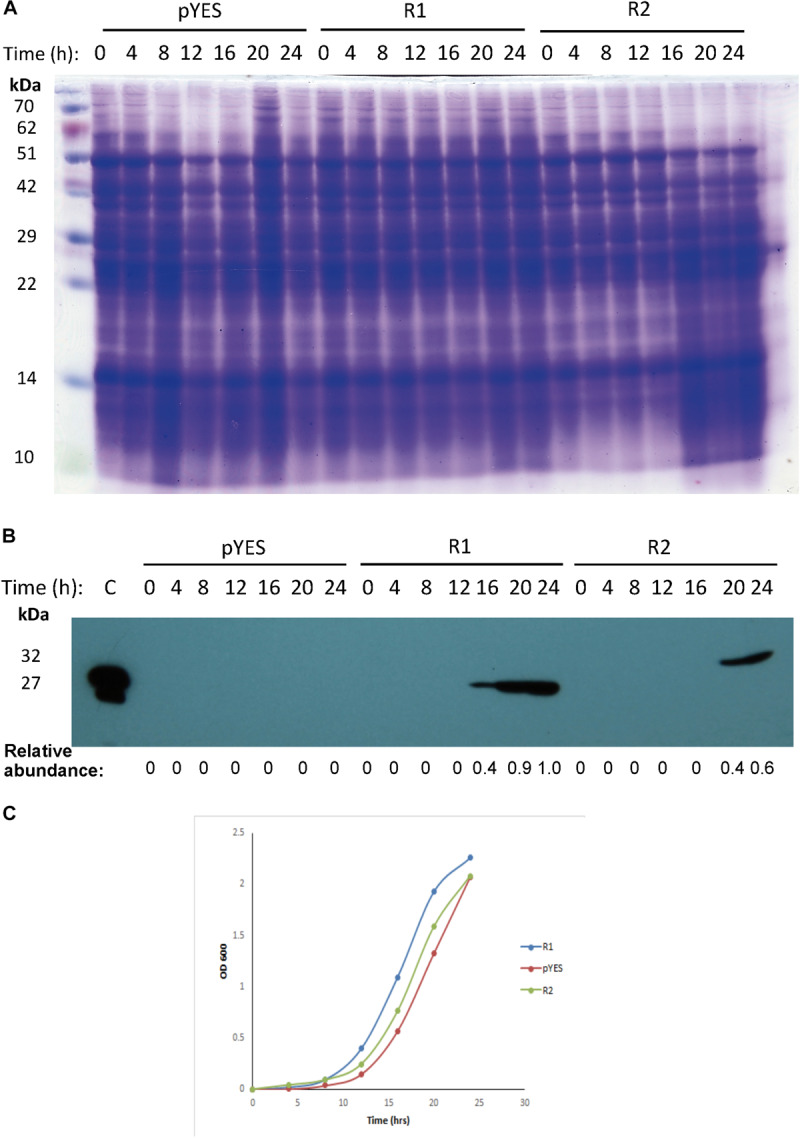
Expression and effect of mayahuelin from *A. tequilana* var. *azul* on growth of *S. cerevisiae* cell cultures. **(A)** SDS-PAGE of yeast total protein extracts from strains transformed with an empty- or a pYES-DEST52 vector expressing *Mayahuelin* gene. Gel shows the protein profile after 0, 4, 8, 12, 16, 20, and 24 h of galactose induction in pYES-DEST52::*Mayahuelin* (R1), pYES-DEST52::*Mayahuelin::V5::6his* (R2) or mock-transformed (pYES) *S. cerevisiae* cells of the W303-a strain. **(B)** Analysis of mayahuelin content in yeast extracts by immunobloting using anti-mayahuelin antibodies. Lane marked **(C)** shows a positive control extract made from spike leaves of *A. tequilana* var. *azul*). **(C)** Growth curves of yeast cells transformed with R1(circles), R2 (triangles) or pYES (diamond). Protein levels shown in b panel are relative to 24 h time point in R1 sample.

To evaluate the cytotoxicity of mayahuelin, two different plasmid constructs were engineered to express the *Mayahuelin* gene in the W303-1a yeast strain: pYES-DEST52::*Mayahuelin* (R1), pYES-DEST52::*Mayahuelin*::*V5*::*6his* (R2) (Supplementary Figure S7). Total protein extracts from transformed yeast cells (Figure 7A) were evaluated after galactose induction for the presence of mayahuelin protein by immunoblot analysis. In R1-transformed yeast extracts, a 27 kDa well-defined band was detected after 16h of galactose induction reaching maximum levels at 24 h (Figure 7B). This band corresponds to mature mayahuelin. Yeast cells transformed with *Mayahuelin*::*V5*::*6his* plasmid (R2) expressed a 32 kDa band (expected molecular mass for mayahuelin fused to *V5* and *6xHis* epitopes) at 20 and 24 h post induction with lower intensities than those observed for R1-transformed yeast (Figure 7B). 24 h growth curves profiles for R1-, R2- and mock- transformed (pYES) yeast were obtained in SD-galactose, plus requirements, induction medium (Figure 7C). Similar growth behavior was observed for all three strains with an exponential phase starting at 12 h that was maintained until 20 h. At 24 h, only pYES cultures upheld an exponential growth pattern, while R1 and R2 showed a slight decrease indicating a probable entrance to post-diauxic shift phase (Figure 7C).

### Mayahuelin Inhibits Luciferase *in vitro* Translation on a Wheat Germ System

**FIGURE 8:**
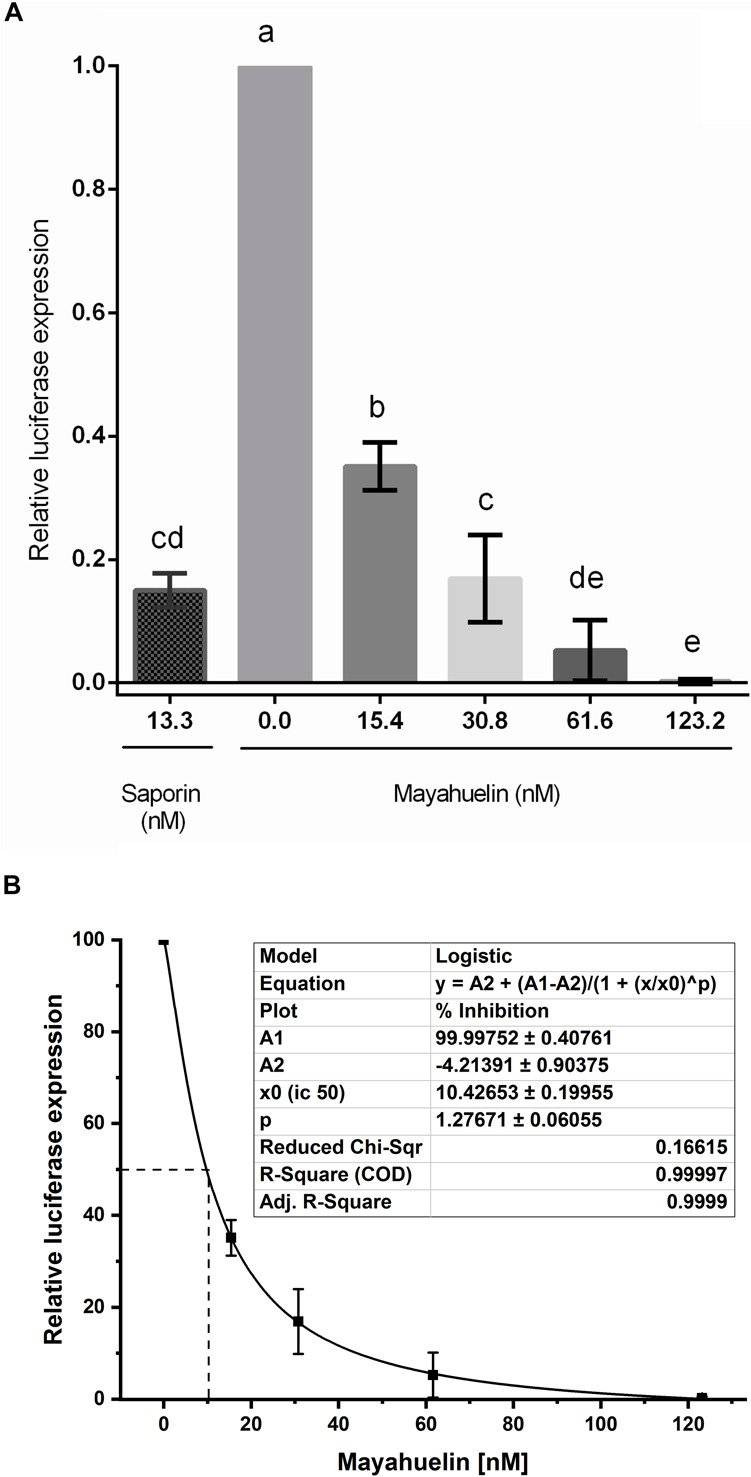
Effect of mayahuelin from *A. tequilana* var. *azul* on protein translation *in vitro* on a wheat germ cell-free system. **(A)** A pure mayahuelin preparation obtained by chromatogaphic methods was assayed on a cell-free wheat germ translation system at the concentrations indicated (0–123.2 nM). A commercial preparation of saporin (from *Saponaria officinalis*) was used at 13.3 nM as a positive control. Luciferase translation was used as a reporter and its activity was measured on a luminometer. Data shown are the mean and standard deviation of three independent experiments. Statistical significant differences between treatments were calculated by one-way ANOVA using *GraphPad Prism v6* software. Bars with different letters are statistically different (*P* < 0.05). **(B)** Mayahuelin dose-response curve on luciferase traslation inhibition. Data from experiments in **(A)** was used to estimate the half-maximal inhibitory concentration (IC_50_) of mayahuelin using Software *Origin v9.6.* Continuous line represents the graph adjustment. Dotted line corresponds to IC_50_.

Mayahuelin was purified to homogeneity using a protocol (Lledías, Gutiérrez, and Nieto-Sotelo, in preparation) based on standard chromatographic methods to directly evaluate its effects on protein synthesis. Mayahuelin inhibited luciferase *in vitro* translation in a dose-dependent manner, when tested on a wheat germ cell-free system. At an initial concentration of 15.4 nM, luciferase translation was 0.65 relative to control with no RIP. At 30.8 nM the registered inhibition was 0.83, a similar value to the 0.85 obtained when saporin was added at 13.3 nM used as positive control. Full inhibition of luciferase translation was obtained when mayahuelin reached 123.2 nM (Figure 8A). The translation inhibitory effects at 30.8- and 61.6 nM mayahuelin were not statistically different to saporin at 13.3 nM (Figure 8A). Luciferase relative expression values were transformed to inhibition percentage and adjusted at a dose-response curve. Mayahuelin showed an IC_50_ = 10.43 nM (*R*^2^ = 0.999) for protein *in vitro* translation in the wheat germ system employed.

### A Phylogenetic Reconstruction of *Agave* Based on *Mayahuelin* DNA Sequences

We used *Mayahuelin* gene as a phylogenetic marker with the following three main goals: to understand whether the Y76D substitution was found in other Agavoideae species, to discern its possible relation to domestication/improvement in the genus, as there is an ancient human history of cultivation and exploitation of wild Agavoideae populations, and to identify close relatives of the cultivated varieties within wild populatons of Rigidae to contribute to their conservation.

**FIGURE 9:**
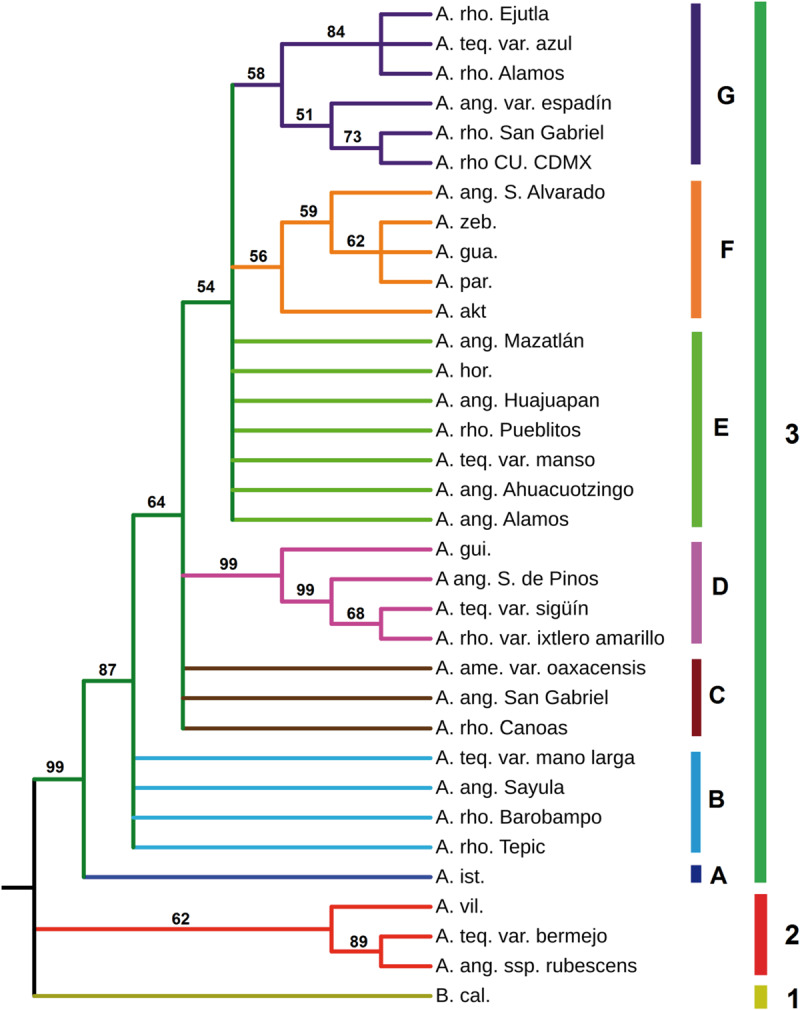
Phylogenetic reconstruction derived from analyses of *Mayahuelin* ortholog gene sequences from Agavoideae. Ortholog mayahuelin sequences (see Supplementary Table S6 and Supplementary Figure S4) were aligned and a phylogenetic reconstruction was obtained based on Bayesian inference algorithms, as described in “Materials and Methods” section. Posterior probability values are given for each branch of the tree. Abbreviations of species names are: A. akt. (*A. aktites*), A.ame. (*A. americana*), A.ang. (*A. angustifolia*), A.gua. (*A. guadalajarana*), A.hor. (*A. horrida*), A.gui. (*A. guiengola*), A.ist. (*A. isthmensis*), A.par. (*A. parryi*), A.rho. (*A. rhodacantha*), A.teq. (*A. tequilana*), A.vil. (*A.* vilmoriniana), A.zeb. (*A. zebra*), and B.cal. (*Beschorneria calcicola*). *Mayahuelin* Genbank accession numbers from all taxa analyzed in figure are indicated in Supplementary Table S6. Words after the species abbreviation refer to either the cultivar (i.e. azul) or the locatity of origin of the specimen (i.e. Alamos). Nucleotide sequence alignment and phylogenetic tree can be found at the TreeBASE website (http://purl.org/phylo/treebase/phylows/study/TB2:S25921).

Phylogenetic reconstructions were obtained by Maximum-likelihood (ML) and by Bayesian inference (BI) methods. Estimates of the ML and BI phylogenetic analyses are shown in Table 2. In ML, GTR + Γ + I was the best nucleotide substitution refinement. As expected for a protein coding marker such as *Mayahuelin*, in BI analyses codon + GTR proved to be the best reconstruction relative to nucleotide-based refinements. As shown in Figure 9 and Supplementary Figure S13, the genetic diversity of *Mayahuelin* within Agavoideae has been sufficiently rapid to grant resolution at the intraspecific, intrageneric, and intergeneric levels. In both ML and BI analyses *B. calcicola* was resolved as a separate lineage (clade 1) from all *Agave* taxa. In BI (Figure 9) two *Agave* lineages were clearly resolved: one represented by *A. vilmoriniana*, *A. tequilana* var. *bermejo*, and *A. angustifolia* ssp. *rubescens* (clade 2), and a very strongly supported second lineage (posteriror probability = 99) including all other species analyzed (clade 3). *A. rhodacantha*, *A. angustifolia*, and *A. tequilana* accessions were polyphyletic, as they were dispersed among the two *Agave* clades in the tree (Figure 9). Clade 2 was the earliest diverging group suggesting a more distant relationship of *A. vilmoriniana*, *A. tequilana* var. *bermejo*, and *A. angustifolia* ssp. *rubescens* relative to the other species in the tree.

**TABLE 2:** Estimates of substitution reconstructions used in Maximum-likelihood and Bayesian inference analyses.

	**Maximum likelihood parameter estimates**	**95% credible interval of Bayesian parameter samples**	
			**Total ESS**
Refinement	GTR + Γ + I	codon + GTR	NI
Llk	−179,358,746	NI	NI
AIC	373,717,492	NI	NI
TL	NI	0.20176–0.27790	4511
r(A<->C)	1.68181	0.15769–0.28965	2379
r(A<->G)	1.94162	0.20164–0.33773	1810
r(A<->T)	0.77428	0.04246–0.13191	2588
r(C<->G)	0.81229	0.07187–0.17619	2573
r(C<->T)	1.21419	0.09647–0.23001	1760
r(G<->T)	1.00000	0.07639–0.19743	2630
pi(A)	0.28822	NI	NI
pi(C)	0.26051	NI	NI
pi(G)	0.25992	NI	NI
pi(T)	0.19135	NI	NI
Alpha	0.72500	NI	NI
Pinvar	0.53100	NI	NI

*Maximum-likelihood estimates for the selected reconstruction were obtained using the ML tree. Credible intervals were obtained from the stationary distribution. Abreviations: Llk, likelihood scores; AIC, Akaike Information Criterion; TL, tree length; r, reversible substitution rate between indicated bases; pi, stationary frequency of indicated base; alpha, shape parameter of the gamma distribution of rate variation; pinvar, proportion of invariable sites; ESS, estimated sample size; NI, parameter not included in selected refinement. For a complete description of the total stationary frequency of codons and the Effective Sample Size (ESS) of Bayesian analyses see Supplementary Table S7.*

The major lineage (clade 3), was subdivided in seven subclades: four of them well resolved (A, D, F, and G) and three of them showing hard polytomy (B, C, and E). Subclade A had a single species (*A. isthmensis*) and its separation from subclades B, C, D, E, F, and G was well supported (posterior probability = 87). Subclade D was very strongly supported (posteriror probability = 99) and revealed *A. tequilana* var. *sigüín* as a close relative of a cultivated form (*ixtlero amarillo*) of *A. rhodacantha* from southern Jalisco, a wild specimen of *A. angustifolia* also from southern Jalisco, and *A. guiengola* from Oaxaca. *A. zebra*, *A. parryi*, *A. guadalajarana*, a wild accession of *A. angustifolia* from Sinaloa, and *A. aktites* conformed the well resolved subclade F, whereas *A. tequilana* var. *azul*, a wild (from Sonora) and one cultivated (from Oaxaca) forms of *A. rhodacantha*, *A. angustifolia* var. *espadín*, a wild *A. rhodacantha* accession from southern Jalisco, and a cultivar of *A. rhodacantha* from unknown origin (UNAM) clustered in clade G. The sub-branch of G subclade that included *A. tequilana* var. *azul, A. rhodacantha* from Alamos, Sonora, and *A. rhodacantha* from Ejutla, Oaxaca was well supported (posterior probability = 84).

Polytomic subclades B and C were composed by *A. rhodacantha*, *A. angustifolia*, and *A. tequilana* accessions in addition to *A. americana* present only in subclade C. Likewise, polytomic subclade E included *A. rhodacantha*, *A. angustifolia*, and *A. tequilana* accessions plus *A. horrida.*

A BI phylogenetic reconstruction that included only *Mayahuelin* sequences from *A. tequilana* and *A. angustifolia* var. *espadín*, showed that the *azul*, *espadín*, and *manso* varieties were the closest relatives, followed by *mano larga* and *sigüín*, whereas *bermejo* is a distant relative of the first five (Figure 9 and Supplementary Figure S9, Supplementary Information).

The ML reconstruction (Supplementary Figure S13) confirmed subclades D, G, and, partially, clade 2 of the BI reconstruction (Figure 9). All other species of clade 3 were unresolved by ML methods. In general, the ML topology had low support values that ranged between 39 and 68 (Supplementary Figure S13); an exception was clade 2, showing a moderate support value (81).

Sequences of the 34 taxa showed high variability at amino acid position 76 of mature mayahuelin, an important residue in the active site of the protein, with six allelic states present: Y/Y, D/D, S/S, Y/D, Y/S, and D/S (Supplementary Figure S10, Supplementary Table S5). Both *A. tequilana* var. *azul* and *A. angustifolia* var. *espadín* were homozygous for the Y76D substitution, confirming their closer relationship relative to other *A. tequilana* varieties (Figure 9; Supplementary Table S5, and Supplementary Figure S9, Supplementary Information).

## Discussion

### Mayahuelin Protein Is Highly Conserved and Active Site Substitution Alleles Are Common in Agavoideae

Mayahuelin from *A. tequilana* var. *azul* is an atypical RIP, as one of the canonical amino acids that compose its active site (tyrosine 76) is substituted by aspartate (Figure 6 and Supplementary Figures S2, S6). A similar natural mutation was reported previously for charybdin, a RIP from *D. maritima*, also a member of the Asparagaceae family (Touloupakis et al., 2006). Here, we found that the frequency of *Mayahuelin* ortholog genes in other Agavoideae species, encoding amino acid substitutions at Y76, is unexpectedly high (Supplementary Figure S10, Supplementary Table S5, Supplementary Information), becoming even more intriguing the study of their physiological or ecological roles in the plant.

Mayahuelin expression in spike leaves of *A. tequilana* var. *azul* is quite high, representing at least 20% of the total protein (Figure 1A). This high level of RIP abundance is comparable to reports in other monocotyledonous plants, as for charybdin and for a type I RIP from *Iris hollandica* (Van Damme et al., 1997; Touloupakis et al., 2006). Both *D. maritima* and *I. hollandica* accumulate RIPs in the bulb, a storage organ where perhaps they could serve as storage proteins, given their large quantities. It is also tempting to speculate that the high levels of accumulation of mayahuelin in immature leaves and mature seeds serve as a storage protein. Expression of vegetative storage proteins is dynamic: they are regulated in response to nitrogen nutrition, wounding, and hervibory (Staswick, 1990; Berger et al., 2002). Thus, the physiological status of the specimens analyzed could explain the disparate levels of mayahuelin in spike leaves (Figure 5) and the lack of correlation with their genetic distance relative to *A. tequilana* var. *azul*. For example, *A. zebra* clusters in clade F, which is a sister group of clade G where *A. tequilana* var. *azul* belongs (Figure 9). However, mayahuelin was not detected in *A. zebra* (Figure 5, lane 14). In contrast, *A. vilmoriniana*, on clade 2 is very distant from *A. tequilana* var. *azul* (Figure 9), but contained very high levels of mayahuelin (Figure 5, lane 13). Alternatively, and not mutually exclusive, levels of mayahuelin could also be the result of the affinity of the mayahuelin primary polyclonal-antibody toward mayahuelins from other species. We found that, as soon as the most developed leaf in the spike unfolds, mayahuelin levels decrease drastically (Figures 1D,E). This evidence supports the role of mayahuelin as a vegetative storage protein. However, we do not exclude additional roles of mayahuelin in defense against hervibory or other regulatory or enzymatic roles (Stirpe and Batelli, 2006; Stirpe, 2013). As discussed forward, the role of humankind during plant domestication or exploitation of plant natural resources for its benefit could also be the driving force for selection of novel structures in the active site of type I RIPs, which by themselves, are not naturally cytotoxic, as they lack the protein (B chain) containing the cell-binding domain for cell internalization, typical of type II RIPs (Stirpe and Batelli, 2006; Stirpe, 2013). Interestingly, type I RIPs can be internalized and strongly enhance its cytotoxic effects when combined with triterpenoid- or steroidal- saponins, that increase membrane permeability (Korchowiec et al., 2015). As steroidal saponins are commonly found in *Agave* (Santos-Zea et al., 2012), this synergic interaction is a possibility worth studying further.

### Activity of Mayahuelin Could Be Affected by Structural Changes in Its Active Site

When mature *Mayahuelin* gene was introduced in *S. cerevisiae* the cells expressed mayahuelin after galactose induction as a 27 kDa protein, in the R1 transformed strain, and as a 32 kDa protein, in R2 cells (Figure 7B). RIP heterologous expression is lethal in yeast when the canonic RIP catalytic site is intact, for example when PAP [type I RIP from *Phytolacca americana*] (Hur et al., 1995) or ricin A chain (Li et al., 2007) are expressed. We found that mayahuelin expression was not cytotoxic in *S. cerevisiae* (Figure 7C). The natural substitution of one tyrosine residue by aspartate in the mayahuelin active site (Y76D) could explain the null cytotoxic effect in yeast cells, although it is also conceivable that other catalytic or non-catalytic domains in mayahuelin are responsible for its null yeast growth inhibition.

Although not as innocuous as in yeast cells, the IC_50_ obtained for mayahuelin on a cell-free wheat germ extract was 10.43 nM (Figure 8). The IC_50_ for inhibition of protein synthesis by RIPs varies according to the cell-free system used (Fuchs, 2019) and seems to be mediated by domains outside the catalytic site, which are highly variable among RIPs. For example, the IC_50_s for a RIP from *Clerodendrum aculeatum* (CA-SRI) are 0.008- and 0.8 nM in rabbit reticulcyte lysate and wheat germ systems, respectively, Kumar et al. (1997). RIP IC_50_s in the wheat germ system cover more than three orders of magnitude, ranging from 0.2 nM, in the case of dodecandrin, to 800 nM for ricin A chain (Harley and Beevers, 1982; Reisbig and Bruland, 1983; Ferreras et al., 1993; Bonnes et al., 1994; Fuchs, 2019). In comparison, mayahuelin classifies as a RIP with medium capacity for protein inhibition in the wheat germ system.

**FIGURE 10:**
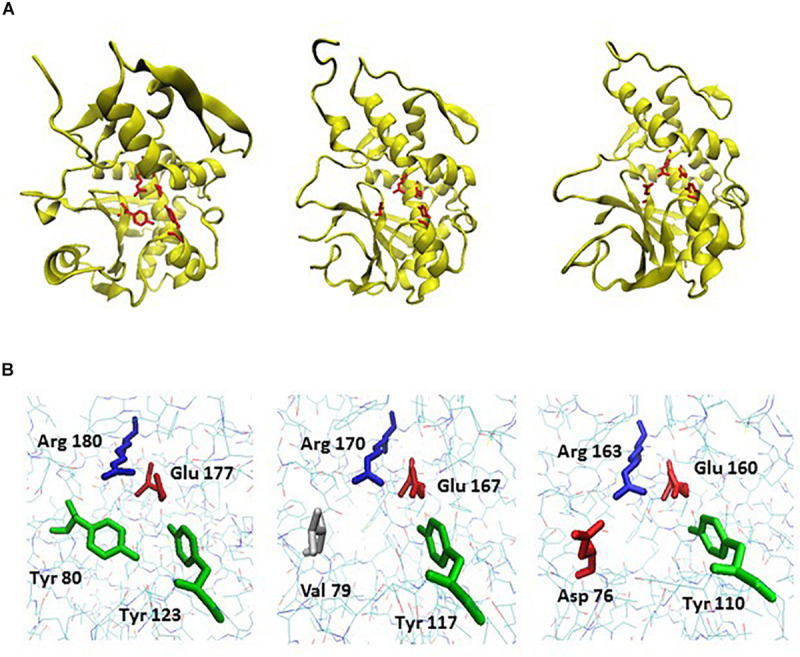
A structural homology model for mayahuelin and its comparison to the crystal structural models of charybdin and ricin. **(A)** Backbone and ribbon diagrams of the proteins showing the side chains of invariable amino acids in their active sites. From left to right: crystal structure of ricin A chain at 1.8 °A [PDB: 1IFT], crystal structure of charybdin at 1.6 °A [PDB: 2B7U], and homology model of mayahuelin from *A. tequilana* var. *azul* (see “Materials and Methods” section). Catalytic amino acids are shown in red. **(B)** Wireframe diagrams of the same proteins focusing on active site region only. From left to right: ricin A-chain, charybdin, and mayahuelin. The position number and name of the four invariable residues of the active site are shown. The.pdb files were obtained from Protein Data Bank [https://www.rcsb.org]. Homology model for mayahuelin was obtained as described in “Materials and Methods” section using charybdin structure as a template. Images were edited with VMD v1.9.3 visualization program.

The low toxicity of mayahuelin *in vivo* and *in vitro* could be related to its active site Y76D substitution. The structural analysis of available RIPs indicate that two tyrosine residues in the active site are aligned in parallel and confine the substrate adenine (Figure 10; Monzingo and Robertus, 1992; Savino et al., 2000). A tyrosine in a homologous region in the ricin catalytic site diminished ricin catalytic activity 15 times, when substituted for phenylalanine [Y80F] (Ready et al., 1991) and the same substitution completely abolished saporin depurination activity (Bagga et al., 2003). Ricin substitution Y80S, of a tyrosine that stabilizes the adenine-substrate, decreases depurination activity by 160-fold (Ready et al., 1991; Kim and Robertus, 1992). A decrease in translation inhibitory activity -about two orders of magnitude compared with ricin- was found in charibdyn, the first reported naturally occurring RIP with an active site mutation (Touloupakis et al., 2006). In the crystal structure of charybdin valine 79 -which substitutes Y79 in the active site- is not aligned with tyrosine 117. As a result, an open conformation of the active site is adopted (Figure 10). In addition, the valine aliphatic chain is not capable to keep the substrate adenine residue in place (Touloupakis et al., 2006). According to our mayahuelin homology-based model, D76 does not align properly with Y110 causing a more open conformation of the active site, as in charybdin (Figure 10). This open conformation may cause an incorrect alignment of the substrate adenine in the SRL of the rRNA, decreasing depurination efficiency. A more complete understanding of the Y76D substitution in mayahuelin should emerge once a mayahuelin with a canonical Y76 version is characterized. This experiment remains pending.

### *Agave* Phylogeny and Evidence of Disjunt Distribution Within Rigidae Group

The three Littaea species studied in this work were distributed in three different clades/subclades. *A. vilmoriniana* formed part of clade 2, with affinities to one *A. tequilana* and one *A. angustifolia* accessions, whereas *A. guiengola* and *A. horrida* were distributed in subclades D and E, respectively (Figure 9). This is in agreement with previous phylogenetic studies performed in Agavoideae (Bogler and Simpson, 1996; Bogler et al., 2006; Good-Avila et al., 2006; Eguiarte et al., 2013) showing the intermixing of species of the Littaeae and Agave subgenera, indicating that they are not monophyletic. However, two members of the Parrynae group (*A. guadalajarana* and *A. parryi*) clustered together and were consistent with their taxonomic classification (Gentry, 1982).

In Mexico, 53 *Agave* species are used for the production of mescal, tequila, and other alcoholic beverages. Out of these, eleven species are intensively cultivated by clonal propagation (Torres et al., 2015) causing an enormous decrease in genetic diversity as more areas are dedicated every year for their cultivation and their wild relatives are continuously extracted from their natural habitat. Identification of the closest wild relatives of the cultivated forms is necessary for the deployment of conservation strategies and sustainable management practices for agave cultivation. More than 15 varieties are known for *A. tequilana* and their origins have been subject to speculation prior to the use of molecular phylogeny approaches. Using morphological characters, Gentry discussed the origin of four cultivars (*azul*, *listado al margen*, *manso*, and *pata de mula*) as well as *A. angustifolia* var. *espadín* from specific wild populations in Mexico (Gentry, 1982) while Valenzuela proposed the identity of four cultivars: *sigüín*, *moraleño*, *bermejo*, and *chato* (Valenzuela Zapata, 1995). More recently, the phylogenetic relationships between nine *A. tequilana* cultivars were derived using AFLP markers (Gil-Vega et al., 2006) recognizing three closely related groups: 1) *azul*, *azul listado*, *sigüín*, *manso*, and *moraleño*, 2) *bermejo*, and 3) *chato*, *hoja delgada*, and *pata de mula*. The relationships between *A. tequilana* cultivars and other *Agave* species, using SSAP of Ty1*-copia* retrotransposons, confirmed the close genetic distance between *azul*, *azul listado*, and *sigüín*, and revealed a close proximity between these varieties with a cultivated form of *A. rhodacantha* (var. *zopilote*), and a more distant relationship to *A. angustifolia*, *A. sisalana*, *A. americana*, and *A. filifera* (Bousios et al., 2007). In a study of three *Agave* species of the State of Jalisco (*A. angustifolia*, *A. tequilana*, and *A. rhodacantha*), where tequila is made, the use of SSR markers revealed that *A. angustifolia* populations from southern Jalisco are close to *A. tequilana* var. *azul*, whereas *A. tequilana* var. *sigüín* and *chato* are close to the cultivar *A. rhodacantha* var. *ixtlero amarillo* (Trejo et al., 2018). In a different study, and using AFLP markers, *A. tequilana* var. *azul* was found in close genetic proximity to *A. angustifolia* var. *espadín* from Oaxaca and to a cultivated form of *A. rhodacantha* also from Oaxaca (Rivera-Lugo et al., 2018). Based on SSR markers, *A. angustifolia* populations also show a large genetic diversity (Trejo et al., 2018).

Our study encompassed the most ample geographical distibution and the largest number of localities for both wild and cultivated accessions from *A. angustifolia* and *A. rhodacantha* used to date to derive *Agave* phylogenies (Rivera-Lugo et al., 2018; Trejo et al., 2018). This ensamble of sequences enabled a more comprehensive analysis of the genetic relationships between the *A. tequila*na varieties to members of the Rigidae group, represented in this work by *A. tequila*na, *A. angustifolia*, *A. rhodacantha*, and *A. aktites*. BI algorithms very strongly support the idea that the Rigidae group is not monophyletic. Clearly, *A. tequilana* var. *azul* is close to *manso* and *sigüín* varieties (Figure 9) and agree with previous studies (Gil-Vega et al., 2006; Bousios et al., 2007). In addition, we found that *mano larga* is next of kin of *azul*, *manso*, and *sigüín*, whereas *bermejo* is very distant and closely related to *A. angustifolia* ssp. *rubescens* (Figure 9 and Supplementary Figure S9, Supplementary Information). The large genetic distance between *bermejo* and other *A. tequilana* varieties was reported earlier (Gil-Vega et al., 2006). Our phylogeny also captured the close proximity between *A. tequilana* var. *azul* and *A. angustifolia* var. *espadín* from Oaxaca found by other authors using morphological or AFLP molecular markers (Gentry, 1982; Rivera-Lugo et al., 2018).

*A. rhodacantha* accessions from Sonora and Oaxaca showed the closest kinship to *A. tequilana* var. *azul* (from the Tequila region in Jalisco) (Figure 9 and Supplementary Figure S4). Relative to *A. tequilana* var. *azul*, the percent identity of *Mayahuelin* gene from the former is 100% whereas that of the latter is 99.72%, although next in proximity to *A. tequilana* var. *azul*, according to BI and ML methods, are a wild *A. rhodacantha* accession from southern Jalisco, an ornamental *A. rhodacantha* of unknown origin (UNAM campus in Mexico City), and *A. angustifolia* var. *espadin* from Oaxaca with percent identities relative to *A. tequilana* var. *azul* of 99.71, 99.71, and 96.65%, respectively. Nonetheless, it is striking the larger genetic distance between *A. tequilana* var. *azul* and its neighboring populations of *A. rhodacantha* from Nayarit and southern Jalisco. Similarly, *A. angustifolia* var. *espadín* from Oaxaca is closer to *A. rhodacantha* accessions from southern Jalisco than those from Oaxaca whereas *A. tequilana* var. *bermejo* is closer to *A. angustifolia* ssp. *rubescens* from Guerrero (Figure 9). This was not the case for *A. tequilana* var. *manso*, *sigüín*, and *mano larg*a that showed close kinship to agaves from southern Jalisco, according to BI (Figure 9). These results contribute to the conservation genetics of agaves used for tequila and mescal production, as they represent the first step toward the genetic identification of natural populations of their wild ancestors. One of the weaknesses of this work resides in the use of only one accession (living specimen) from each population analyzed, which does not allow to assess their genetic diversity. Therefore, future studies on the origin of domesticated agaves should include a larger number of individuals from each of these populations to have a more statistically significant estimation of their identity relative to their closest wild relatives. The domesticated alleles could be rare alleles in other wild populations that, on the other hand, may display similarities at different levels of complexity. Furthermore, it is believed that the success of the genus *Agave* is due in part to the high frequency of polyploid species within the group. Agaves have a basic chromosome number = 30 and euploid series of 2n, 3n, 4n, 5n, and 6n have been reported (Granick, 1944). Thus, the reconstruction of *Agave* phylogenies based on few or single genetic markers, such as *Mayahuelin*, could underestimate their full ancestry, especially in cases where allopolyploid speciation events occurred. Therefore, our results are indicative, but no fully conclusive, of the origin of *A. angustifolia*, *A. tequilana*, and *A. rhodacantha* cultivars.

A full understanding of the disjoint distributions in the phylogeographic pattern between these cultivars and their closest wild relatives requires both ecological and cultural elements for discussion. Although bats are long-distance flyers and agave seed set is dependent on bat pollination to achieve full potential (Howell and Roth, 1981) agave seed dispersal depends mostly on wind and water (Sánchez-Salas et al., 2017; Lindsay et al., 2018). During the warmest half of the year dominant winds in Mexico move with a NE to SW direction. During the coldest half of the year dominant winds in northern Mexico blow from the W, while maintaining a NE to SW direction in southern Mexico and occasionally receiving northern winds from the Gulf of Mexico (García, 2003). Thus, prevailing wind patterns in Mexico would not favor connectivity between Sonora, Jalisco, and Oaxaca. However, from a theoretical perspective, wind dispersal of agave seed from Oaxaca to Sonora, but not the opposite, is possible since, during the summer and autumm, hurricanes run parallel to the coasts of Mexico moving on a SE to NW direction, ocassionaly entering inland (García, 2003; Rosengaus Moshinsky et al., 2002). Thus, it is theoreticaly unlikely the dispersion of agave seed from Sonora to Oaxaca, especially if we consider the large geographical distance between these states (Supplementary Figure S1). Assuming long distance wind dispersal of agave seeds, germination and establishment of agave seedlings in natural habitats is extremely low (Nobel, 1992; Arizaga and Ezcurra, 2002) making even more difficult to explain the observed disjoint distribution by wind seed dispersal. Alternatively, disjoint distributions and presence of plants far outside their natural range can be explained if we consider the long history of plant cultivation by human cultures. Because of their small size and weight, *Agave* seeds and bulbils could have been dispersed by humans along ancient trade routes. Such long distance trade was intense in Mesoamerica (present day Mexico and Guatemala) and North America (i.e. Mississippian culture of southeastern and midwestern United States) from around 1600 BCE and between 1000 and 1550 CE, respectively, Smith (2010). Moreover, cultural connections between Mesoamerica and Sonora exist since prehispanic times (Watson and García, 2016) and pinpoint Western Mexico (Jalisco, Nayarit, Colima, and Michoacán) as a macro-regional economy connecting Mesoamerica and the United States Southwest by both coastal and highland routes (Wilcox et al., 2008). Evidence for diffusion of plants along these routes is better understood in maize, whose introduction to the United States Southwest from Mesoamerica began around 2000 BCE, via the highland route, and 2000 years ago, via the coastal route (da Fonseca et al., 2015). In the case of some *Agave* species, ancient traces of their cultivation by Native Americans have been recognized based on genetic structure and the patchy distribution of colonies typically found in close proximity to prehistoric settlements (Minnis and Plog, 1976; Gentry, 1982; Parker et al., 2010; Lindsay et al., 2018). Historical accounts report the movement of *A. salmiana* and *A. americana* from central- (Tlaxcala) to northern- Mexico (Saltillo and Durango), right after the conquest of Mexico by the Spaniards, as thousands of náhuatl-speaking peoples, mainly Tlaxcaltecs, colonized the region bringing with them *maguey* to maintain their deep-rooted tradition of *pulque* production (Gentry, 1982). During the XVII century (*circa* 1621) agaves were already under cultivation in the Tequila region of Jalisco (Valenzuela Zapata, 1995) although historical accounts of their origin are still unknown. It remains to be studied how *A. angustifolia* var. *espadin*, *A. tequilana* var. *azul* and *A. rhodacantha* from Alamos, Sonora are interconnected and a more in depth study of natural populations of the Rigidae group in the Pacific coast of Mexico is needed.

Variation of some characters within the *A. angustifolia complex* is so large that separation at the species level is very difficult (Gentry, 1982). Our work has provided evidence for the ample genetic diversity within the *A. angustifolia* complex (Figure 9) and agrees with previous studies (Rivera-Lugo et al., 2018). We have also provided novel evidence for the large genetic diversity within the *A. rhodacantha* complex and the polyphyletic origin of Rigidae whose members are distributed in seven subclades/clades (Figure 9).

The *A. angustifolia* complex has the most ample distribution of agaves in North-America: inhabits diferent plant communities (thorn forests, tropical savannah, and drought-deciduous tropical forests) between sea level to near 1,600 m. a.s.l. It is possible that the long, narrow, rigid leaves typical of *A. angustifolia* and *A. rhodacantha* represent an ecomorphological response of different species to similarities in microenvironmental conditions across their distribution range (i.e. aridity, dew formation at night, etc.), causing confusion on the identification of phylogenetic signals based on pure morphological characters. The phenotypic similarity observed across independent lineages of closely related species has lead to the concept of phylogenetic niche conservatism (PNC) (Bravo et al., 2014). PNC has been observed in different taxa of the plant and animal kingdoms (Losos, 2008) and the *A. angustifolia* and *A. rhodacantha* complexes could be experiencing this process. In agaves and other xerophytic rosette plants, long-narrow leaf morphologies are very efficient for fog-harvesting (Martorell and Ezcurra, 2007). It remains to be studied if, in the *A. angustifolia* and *A. rhodacantha* complexes, such traits relate to their ecological niches along the Pacific and Atlantic coasts of Mexico and Central America. The phenotypic similarity could be the result of similar changes at the genetic level, as leaf growth and morphogenesis proceed through conserved genetic mechanisms (Nelissen et al., 2016). However, it can be due to other mechanisms such as gene flow among taxa, genetic drift, etc. (Bravo et al., 2014). Gentry (Gentry, 1982) also pointed out the dificulty of delimiting species between *A. angustifolia* and some A. *rhodacantha* populations due to overlaps in leaf characters (length, width). As these morphological characters seem to offer little taxonomic value within the Rigidae group in *Agave*, the finding of new diagnostic characters at different levels of complexity, among them DNA sequence markers, and a new taxonomic revision, are pending.

Finally, it is quite evident the hard polytomy at different levels of the tree from a large number of of *A. rhodacantha* and *A. angustifolia* accessions studied (Figure 9). Further work is needed to asses if this polytomy is an artifact caused by the lack of phylogenetic resolution of the genetic marker used or whether it represents a true phylogenetic radiaton pattern due perhaps to the great degree of artificial selection and geographical movement caused by *Homo sapiens*.

#### Possible Causes for Active Site Substitutions in Mayahuelin Within Agavoideae

Are substitutions in the active site of mayahuelin orthologs within Agavoideae the result of domestication, functional specialization or simple evolutionary adaptations for defense against particular viruses, microbes or hervibores? The evidence provided to date is too preliminary to answer with certainty any of these questions. Out of the 34 taxa studied, 13 are cultivated and 21 come from wild populations. The frequency for both Y/Y homocigocity and homozigotic substitutions (D/D and S/S) at aa position 76 was higher in wild accessions relative to cultivars (Supplementary Figure S10 and Supplementary Table S5). In contrast, heterozigocity at position 76 (Y/D, Y/D, and Y/S) was more frequent in cultivars (Supplementary Figure S10 and Supplementary Table S5). Examples of this bias between the Y/Y homocigocity at position 76 and species that have no record of cultivation or human utilization are *A. horrida* and *A. guiengola* as well as wild forms of cultivated species such as *A. angustifolia* and *A. rhodacantha*. Other taxa with a long history of cultivation or utilization as a natural resource since historical or pre-historical times displayed homocigous D/D (*A. parryi, A. vilmoriniana*, and *A. zebra*) or S/S (only found in *A. angustifolia* ssp. *rubescens*) substitutions. The only exceptions of cultivated forms with canonical Y/Y homocigocity at position 76 are *A. rhodacantha* var. *ixtlero amarillo* and *A. tequilana* var. *mano larga* that are used for fiber and mescal production, respectively. In opposition, *A. isthmensis* is a wild species with no history of cultivation or utilization by human beings and is heterozygous for Y/D76. The evolutionary significance of these substitutions should become more clear once a comparative functional study of mayahuelin proteins containing each of the allelic variations is performed.

Our observations could raise the interest in Agavoideae to become great models for in-depth studies on the evolution and functional/structural analysis of RIPs. Moreover, *Mayahuelin* sequences are promising to reconstruct reliable phylogenies within Agavoideae and they could complement information derived using other genetic markers, such as the chloroplast genes or *ITS* sequences used so far.

## Data Availability Statement

The datasets presented in this study can be found in online repositories. The names of the repositories and accession numbers can be found in Figure 6 (mass spectrometry) and Figure 9 (phylogenetic information) of the article as well as in Supplementary Tables S4, S7 of Supplementary Material (DNA sequences).

## Author Contributions

FL identified and developed purification protocols for the biochemical characterization of mayahuelin and for the isolation of anti-mayahuelin antibodies and prepared figures under supervision of GC. SR, JG, and ES designed and performed all experiments for evaluation of the cytotoxic effects of mayahuelin in yeast. JG and JN-S carried all phylogenetic analyses, performed homology modeling, prepared figures and tables, and went on field trips for Agave collection. JG also evaluated effects of mayahuelin on wheat germ system under supervision of TD. FH-B evaluated levels of mayahuelin protein in tissues from different Agave species and prepared figures. AM-H constructed and characterized EST libraries from *A. tequilana* and evaluated mayahuelin transcript levels by RT-PCR. AG-M is the curator of the Agavaceae Collection of IB-UNAM Botanical Garden that provided most Agave tissues, went on field trips, and helped in their taxonomic identification. JN-S conceived the project, analyzed data, supervised all author’s work, and wrote the texts. All authors contributed to the manuscript.

## Conflict of Interest

The authors declare that the research was conducted in the absence of any commercial or financial relationships that could be construed as a potential conflict of interest.

## Footnotes

1 https://blast.ncbi.nlm.nih.gov

2 http://www.rcsb.org

3 http://services.mbi.ucla.edu/ERRAT

4 http://molprobity.biochem.duke.edu

5 http://www.atgc-montpellier.fr/phyml/
