# Supplementary material for: Mayahuelin, a Type I Ribosome Inactivating Protein: Characterization, Evolution, and Utilization in Phylogenetic Analyses of Agave
Source: Front Plant Sci. 2020 May 27;11:573. doi: 10.3389/fpls.2020.00573 (PMC7266874; doi:10.3389/fpls.2020.00573)
Supplement: Supplementary file 1 [file Data_Sheet_1.pdf]

SUPPLEMENTAL INFORMATION SECTION

manuscript:

Mayahuelin, a type I Ribosome Inactivating Protein: characterization, evolution, and utilization in phylogenetic analyses of *Agave.*

Authors:

Fernando Lledías, Jesús Gutiérrez, Aída Martínez-Hernández, Abisaí García-Mendoza, Eric Sosa, Felipe Hernández-Bermúdez, Tzvetanka D. Dinkova, Sandi Reyes, Gladys I. Cassab, and Jorge Nieto-Sotelo

Published in 2020 in:

Front. Plant Sci. doi: 10.3389/fpls.2020.00573

SUPPLEMENTAL FIGURE LEGENDS

Supplementary Figure S1. A map of Mexico showing the locations of taxa studied in this work. In the map, the *A. guadalajarana* (open diamond) position is coincident with that of an *A. rhodacantha* specimen (black star) in northern Jalisco. Likewise, the locations of one specimen of *A. angustifolia* and one of *A. rhodacantha* are very close to each other in southern Jalisco.. Inset: an enlargement of the Nayarit-Jalisco region of Mexico.

Supplementary Figure S2. Alignment of *Agave tequilana* var. *azul* RIP protein family sequences. RIP family member sequences were mined from an *A. tequilana* var. *azul* EST library (Martínez-Hernández et al., 2010; Simpson et al., 2011). Consensus cDNA sequences were constructed and translated *in silico*. Amino acid sequences were aligned with the T-Coffe program. Green arrows point to the highly conserved catalytic amino acid residues found in all RIP family members. Genbank accession numbers for *A. tequilana* var. *azul* RIP sequences are given in Table S4.

Supplementary Figure S3. Cladogram of *Agave tequilana* var. *azul* RIP family members. The nucleotide sequences from *Agave tequilana* var. *azul* RIP family were aligned using *Translator X* and *MUSCLE* programs. After alignment, a phylogenetic analysis based on Bayesian inference with MrBayes v3.2 (Ronquist et al., 2012) was used. The Genbank accession numbers of *A. tequilana* var. *azul* cDNAs encoding RIP family members are shown in Table S6. Numbers along the branches of the tree indicate posterior probability values.

Supplementary Figure S4. Screening for ortholog *Mayahuelin* sequences by phylogenetic analysis. Sequences from each Agavoideae species studied, obtained after PCR amplification of cDNA or genomic DNA with F2-R2 or F6-R6 primer combinations (See "Amplification of *Mayahuelin* orthologs" section in Supplementary Information), were subjected to multiple sequence alignment with the whole *A. tequilana* var. *azul* RIP family. A consensus tree was estimated by the Bayesian inference approach using MrBayes v3.2 (Ronquist et al., 2012) as described in Materials and Methods section. Posterior probability values were calculated for each branch. *Mayahuelin* orthologs were defined as those sequences clustering to aC630_3 clone (highlighted in green), encoding *Mayahuelin*. Sequences clustering to other RIP family members were considered as *Mayahuelin* paralogs. In this example, all sequences tested were orthologs to *Mayahuelin* with the exception of the *A. marmorata* sequence (highlighted in yellow) that clustered with aC2762_3 clone. Abbreviations of species names are: A. akt. (*A. aktites*), A.ame. (*A. americana*), A.ang. (*A. angustifolia*), A.gua. (*A. guadalajarana*), A.hor. (*A. horrida*), A.gui. (*A. guiengola*), A.ist. (*A. isthmensis*), A.par. (*A. parryi*), A.rho. (*A. rhodacantha*), A.teq. (*A. tequilana*), A.vil. (*A.* vilmoriniana), A.zeb. (*A. zebra*), and B.cal. (*Beschorneria calcicola*). *Mayahuelin* Genbank accession numbers from all taxa analyzed in this figure are indicated in Table S6 (with the exception of the *A. marmorata* sequence). Words after the species abbreviation refer to either the cultivar (i.e., azul) or the locatity of origin of the specimen (i.e., Alamos).

Supplementary Figure S5. Prediction of a signal peptide and its subcellular localization at the N-terminus of mayahuelin precursor protein. **A)** The full amino acid sequence of mayahuelin precursor, derived from cDNA clone aC630-3 (Fig. 6), was analyzed with the SignalP-5.0 program (http://www.cbs.dtu.dk/services/SignalP/) available at the ExPASy

bioinformatics portal (https://www.expasy.org/resources/search/keywords:signal%20peptide). Only the first 48 aa are shown in the graph. A cleavage site was predicted between aa positions 22-23 (AAA-DG; *P* = 0.5118). Red line indicates the predicted secretory signal peptide score *Sp (Sec/SPI*) = 0.9759; green line indicates the predicted cleavage site score; orange line indicates the probabiity of not having a signal peptide. **B)** Subcellular localization analysis of mayahuelin precursor using DeepLoc-1.0 (http://www.cbs.dtu.dk/services/DeepLoc-1.0/index.php) available at ExPASy. Panel shows a tree depicting the prediction for subcellular localization using a neural network algorithm trained on Uniprot proteins with known subcellular localization. An extracellular localization signal was predicted at the N-terminus of mayahuelin precursor (likelihood = 0.9577) as well as the soluble nature for the protein (likelihood = 0.9966).

Supplementary Figure S6. Sequence alignment of mature mayahuelin protein from *A. tequilana* var. *azul* and representative plant RIPs. Green arrows and green squares indicate the highly conserved amino acids that conform the catalytic site in RIPs, which, relative to mayahuelin sequence, are: D76, Y110, E160, and R163. Aligment was obtained by using the Clustal Omega algoritm (https://www.ebi.ac.uk/Tools/msa/clustalo/) and RIP Fasta sequences retrieved from NCBI (https://www.ncbi.nlm.nih.gov). Mayahuelin: mayahuelin (GenBank: MN913554) from *A. tequilana* var. *azul* [Asparagaceae]; Charybin: charybdin (PDB: 2B7U-A) from *Drimia maritima* [Asparagaceae]; Ricin_chainA: ricin A chain (PDB: 1RTC_A) from *Ricinus communis* [Euphorbiaceae]; Abrin_chainA: abrin a A chain (GenBank: CAA54138.1) from *Abrus precatorius* [Fabaceae]; Saporin, saporin S6 (GenBank: CAA48889.1) from *Saponaria officinalis* [Caryophyllaceae]; PAP, pokeweed antiviral protein PAP-S (GenBank: 1APA_A) from *Phytolacca americana* [Phytolaccaceae]; and Trichosantin: trichosantin (Genbank: AAA34207.1) from *Trichosantes kirilowii*

[Cucurbitaceae].

Supplementary Figure S7. Schematic structure of yeast expression vectors pYES-DEST52::*Mayahuelin* and pYES- DEST52::*Mayahuelin::V5::6His* used in this work.

Supplementary Figure S8. Position of primers within the *Mayahuelin* gene. All primers used in this work can be visualized relative to the *Mayahuelin* gene from *A. tequilana* var. *azul*. F1 and F2 primers (yellow box), F6 primer (green line), Fwd3_630 primer (blue box). Reverse primers are the reverse complement of green box (Rev3_630 primer), orange line (R6 primer), and gray box (R1 and R2 primers).

Supplementary Figure S9. Phylogenetic relationships of *A. tequilana* cultivars using *Mayahuelin* ortholog genes as molecular markers. *Mayahuelin* nucleotide sequences from five *A. tequilana* and from one *A. angustifolia* cultivars were aligned using the *TranslatorX* and MUSCLE algorithms. *Mayahuelin* from *B. calcicola* was used as outgroup. The phylogenetic reconstruction shown was obtained by the Bayesian Inference method with *codon* option (see Materials and Methods section). Numbers along the branches of the tree indicate posterior probability values.

Supplementary Figure S10. Frequency of allelic states at position 76 of mayahuelin in wild and cultivated *Agave*. *Mayahuelin* ortholog sequences from thirteen cultivars and twenty one wild accessions of the genus *Agave* were analized to deduce the allelic state at position 76 of the mature protein (for a detailed list of all accessions analysed see Table S5). Six allelic states were documented: three homozygous (Y/Y, D/D, and S/S) and three

heterozygous (Y/D, Y/S, and D/S). Bars indicate the percentage of cultivated or wild taxa found under each category.

Supplementary Figure S11. Cladogram of *Agave tequilana* var. *azul* RIP family members using Maximun-likelihood algorithm as described in Materials and Methods section. Numbers along the branches of the tree indicate support values.

Supplementary Figure S12. Screening for ortholog *Mayahuelin* sequences by phylogenetic analysis using Maximun-likelihood algorithm. A reconstruction was obtained using the program *PhyML* as described in Materials and Methods section. Numbers along the branches of the tree indicate support values.

Supplementary Figure S13. Phylogenetic reconstruction derived from analyses of *Mayahuelin* ortholog gene sequences from Agavoideae using Maximun-likelihood algorithm as described in Materials and Methods section. Numbers along the branches of the tree indicate support values.

SUPPLEMENTAL MATERIALS AND METHODS.

Growth conditions and sample preparation for total protein extraction

Conditions for plant growth, tissue collection, and total protein extraction procedures are described in the Supplementary Information section.

Agave tequilana var. *azul* plants were grown in a greenhouse under natural photoperiod with maximal day temperature of 32 °C and minimal night temperature of 20 °C. Plants were watered once every 2 weeks. Other *Agave* species studied here were grown outdoors as part of the public living collection of the Botanical Garden. Mayahuelin was extracted from the basal 15 cm segment of the spike in all *Agave* species studied. For *A. tequilana* var. *azul* experiments that compared mayahuelin organ-specific expression in rosette leaves, stem, and roots, 2 to 2.5 year old plants were used; samples from the spike, internal, middle or outer rosette sectors were taken as described previously (Luján et al., 2009). Samples from peduncle, anthers, filaments, pistils, tepals, and seeds were taken from an *A. tequilana* var. *azul* plant (6-9 year-old) with a fully developed inflorescence grown as an ornamental on UNAM's campus. After collection and slicing, samples were immediately frozen in liquid nitrogen and kept at −70 °C prior to grinding using either dry ice pellets and a commercial coffee grinder or liquid nitrogen and a mortar. Total protein extracts from *Agave* specimens were obtained according to a published protocol specifically designed for the electrophoretic analysis of proteins from succulent plants (Lledías et al., 2017a; Lledías et al., 2017b).

Molecular biology protocols

Mayahuelin* gene cloning and expression in *S. cerevisiae

Mayahuelin total RNA from spike leaves of *A. tequilana* var. *azul* (Luján et al., 2009) was used as template in a cDNA synthesis reaction. cDNA products were amplifiied by PCR using F1 and R1 primers, or F1 and R2 primers, plus Phusion High Fidelity DNA Polymerase (Finnzymes) (See **Supplementary** Table S3 for primer sequences). F1-R1 and F1-R2 products were cloned in pENTR/D-TOPO vector (Invitrogen) and transformed into DH5α *E. coli* competent cells. Plasmid DNA from positive clones was recombined into yeast expression vector pYES-DEST52 (Invitrogen). F-R1 clones (pYES-DEST52::*Mayahuelin*) only express Mayahuelin coding sequence in mature form preceded by a 6 bp *S. cerevisiae* consensus sequence for translation initiation sites followed by an ATG codon and a TGA stop codon at the end of the coding sequence, whereas F-R2 (pYES-DEST52::*Mayahuelin*::*V5*::*6his*) encode *Mayahuelin* fused to V5 epitope and a histidine tag. Both constructs are located downstream of a *GAL-1* inducible promoter (**Supplementary** Fig. S7). F-R1 and F-R2 plasmids were sequenced employing T7 promoter primer (5' TAA TAC GAC TCA CTA TAG GG 3') and a reverse primer (5' CTC CTT CCT TTT CGG TTA GA 3'). F-R1 and F-R2 plasmids with the correct orientation and sequences were introduced into *S. cerevisiae* W303-1a strain (*MAT***a** *can1-100 ade2-1 his3-11,15 leu2-3,112 trp1-1 ura3-1*) according to Gietz and Woods (2002). Resuspended yeast pellets were plated on SD glucose Ura^- solid medium (0.67% yeast nitrogen base without amino acids, 2% glucose, 40 mg/L adenine hemisulfate, 20 mg/L histidine, 20 mg/L tryptophan, and 90 mg/L leucine) and incubated for 72 h at 30 °C. Presence of *Mayahuelin* gene was determined by colony PCR employing F1 and R1 or F1 and R2 primers accordingly. Colonies containing F-R1 or F-R2 constructs were grown overnight on liquid medium (SD-glucose + ade + trp + his + leu) and used to inoculate a flask containing 120 ml of medium at an OD_{600}= 0.05. Cultures were incubated at 25 °C (130 rpm) until an

OD_{600}= 0.4 was reached. Cultures were centrifuged (1,500 X g /4 min at 4 °C) and pellets washed (2X) with sterile water; pellets were centrifuged again and resuspended in 1 mL of SD- 2% galactose + ade + his + trp + leu medium to inoculate 119 mL of fresh medium of the same composition. Cultures were incubated at 25 °C for 24 h at 130 rpm. Culture growth was monitored by absorbance at OD_{600nm} 15 mL aliquots were taken at 0, 4, 8, 12, 16, 20 and 24 h, centrifuged, and pellets resuspended in 100 µl Laemmli 1X sample buffer (60 mM Tris-HCl pH 6.8, 10 % SDS, 2 % [v/v] glycerol, and 5 % [v/v] 2-β-mercaptoethanol). After boiling for 10 min, and centrifugation, 20 µl aliquots from the supernatants were analyzed by western blot to determine mayahuelin levels.

Mayahuelin* transcript accumulation in *A. tequilana* var. *azul.

Isolation of RNA to estimate *Mayahuelin* transcript levels in leaves from different sectors of the rosette of *A. tequilana* var. *azul* was performed as described (Luján et al., 2009). *Mayahuelin* Rev3_630 primer (see **Supplementary** Table S3) was used to reverse transcribe 1 µg of DNAse-treated total RNA with SuperScript reverse transcriptase, according to the manufacturer's protocol (Invitrogen, cat. 18064-014). To determine the concentration of *Mayahuelin* transcripts a calibration curve was made; a plasmid containing the *Mayahuelin* full-length cDNA (Martínez-Hernández et al., 2010; Simpson et al., 2011) was purified with a QIAprep Spin Miniprep kit (Qiagen, cat. 27104) and its concentration was determined with a Nanodrop 1000 (Thermo Scientific). Ten-fold serial dilutions of a 0.1 pg plasmid preparation and of *Mayahuelin* cDNAs obtained from each leaf sample were amplified in triplicate with IQ SYBRGreen Supermix (BioRad, cat. 170-8880) using 20 pmol of Fwd3_630 and Rev3_630 primers (**Supplementary** Table S3). A total of 35 cycles were carried out with the following parameters: denaturation step for 30 s at 95°C, annealing for 30 s at 64°C, and extension for 30 s at 72°C. Initial denaturation and final extension were performed for 5 min at 95°C and 72°C, respectively. Controls without

template were also performed (NTC). The threshold cycles (Ct) were determined with the iQ5 Optical System Software 2.0. The calibration curve was plotted according to a published protocol (Heid *et al*., 1996) and was used to calculate the *Mayahuelin* mRNA quantity in pg present on each sample. Final results were expressed as pg of *Mayahuelin* transcript per µg of total RNA.

Amplification of *Mayahuelin* orthologs in Agavoideae by RT-PCR and by PCR of genomic DNA and DNA sequencing.

Isolation of total RNA from spike leaves of different *Agave* species to amplify *Mayahuelin* ortholog sequences by RT-PCR was done by the Trizol method (Chomczynski and Sacchi, 1987). cDNA products or genomic DNA were amplified by PCR using F6 and R6 primers (**Supplementary** Table S3) and following the protocol described in Suppl. Information. Total genomic DNA from Agavoideae leaves was obtained using a standard protocol (Doyle and Doyle, 1987). PCR products were purified by electrophoresis on 1% agarose gels using Wizard SV Gel and PCR Clean-Up System (Promega, cat. A9280). *Mayahuelin* PCR products were sequenced at the *Laboratorio de Secuenciación Genómica de la Biodiversidad y de la Salud* of Instituto de Biología, UNAM or at *Unidad de Síntesis y Secuenciación de ADN* of Instituto de Biotecnología, UNAM. Sequences were curated with UGENE V1.33 software.

Amplification of *Mayahuelin* genes by PCR

Reactions were prepared according to the following procedure:

PCR reaction mix for amplification of *Mayahuelin* gene from Agavoideae

Reagent	Final concentration
Nucelase-free $H_2$0	to 50 µL
5X Phusion HF Buffer (Thermo Scientific™)	1X
dNTP mix	200 µM each

Forward primer*	0.5 µM
Reverse primer*	0.5 µM
Phusion High-Fidelity DNA Polymerase (Thermo Scientific™ 2.0 U µL^{-1})	0.02 U µL^{-1}
Genomic DNA template	400 ng

*Two different primer sets were used. The F2-R2 primer combination produced 735 bp amplicons. The F6-R5 primer combination generated 720 bp products. Primer sequences are found in **Supplementary** Table S3.

Reactions proceeded on an *Eppendorf* thermal cycler [*Mastercycler gradient*] using the following programs:

	**F2-R2**			**F6-R6**		
**Step**	**T (°C)**	**time**	**# cycles**	**T (°C)**	**time**	**# cycles**
Initial denaturation	98	2 min	1	98	2 min	1
Denaturation	98	10 s	30	98	10 s	35
Annealing	55.5	30 s		59	30 s	
Extension	72	45 s		72	45 s	
Final extension	72	7 min	1	72	7 min	1

Amplification of *Mayahuelin* gene by RT-PCR

Some Agavoideae species yielded products whose nucleotide sequences were unreadable due perhaps to amplification of more than one RIP gene. In such cases, RNA was isolated from spike leaves to serve as template on a cDNA synthesis reaction. Prioir to cDNA synthesis, the RNA sample (1 µg) was incubated with DNAse I (Invitrogen, cat. 180068-015) as follows:

**Reagent**	**Volume**
Total RNA	1 µg
10X buffer DNAse I	1 µL
DNAse I (1 U/µL)	1 µL
Nuclease-free water	to 10 µL

Reaction was kept at room temperature for 15 min. DNAse I was inactivated by addition of 1 µL 25 mM EDTA and maintaining the tube at 65 °C for 10 min.

cDNA synthesis was carried with "SuperScript II" (Invitrogen, cat. 18064-022) reverse transcriptase in the following reaction mix.

Reagent	Volume
DNAse I-treated RNA	1 µg
Oligo dT (500 µg/mL)	1 µL
dNTP mix (10 mM each)	1 µL
Nuclease-free water	to 12 µL

The reaction mix was incubated at 65 °C for 5 min and the following reagents were added:

Reagent	Volume
5X first-strand buffer	4 µL
0.1 M DTT	2 µL

Reaction was transferred to 42 °C for 2 min before adding 1 µL of reverse transcriptase (200 U) and further incubating at 42 °C for 50 min. Enzyme activity was killed at 70 °C for 15 min. RNA template was destroyed by adding 1 µL RNase H (2 U) and incubating at 37 °C for 30 min.

In addition to the experimental samples, a negative control sample was prepared for each one by RNAse treatment prior to the DNAse I treatment described above.

A 5 µL aliquot of the cDNA synthesis reaction provided the template for PCR amplification by using the following protocol:

RT-PCR reaction mix for amplification of *Mayahuelin* gene from Agavoideae:

Reagent	Final concentration
Nuclease-free water	to 50 µL
5X Phusion HF Buffer (Thermo Scientific™)	1X
dNTP mix	200 µM each
Forward primer*	0.5 µM
Reverse primer*	0.5 µM
Phusion High-Fidelity DNA Polymerase (Thermo Scientific™ 2.0 U μL^{-1})	0.02 U μL^{-1}
cDNA (from step above)	5 µL

*The primer combination F6-R5 was used yielding 720 bp products. Find primer sequences in **Supplementary** Table S3.

Reactions proceeded on an *Eppendorf* thermal cycler [*Mastercycler gradient*] using the program recommended for the F6-R6 combination of primers described above.

Expected sizes of PCR products when using genomic DNA from *A. tequilana* var. *azul* as template:

Primer combination	Product size (bp)
F1 + R1	754
F1 + R2	751
F2 + R2	735
F6 + R6	720

Methods for biochemical analysis

Native Gel Electrophoresis

Supernatant samples containing 500 mg protein were loaded onto preparative polyacrylamide native 8 X 6 cm minigels (8% final concentration gels prepared from acrylamide:bis-acrylamide 32:1 stock, 126 mM Tris pH 8.8) and run at 6 ^{o}C for 3 h at 200 V with 24.8 mM Tris and 192 mM glycine as tank buffer. One cm vertical strip of the gel was cut, and proteins were stained with coomassie blue to localize the mayahuelin band that was used as pattern to excise the band out from the original preparative gel.

Native protein electroelution

Mayahuelin containing gel fragment was cut in small cubes (1 mm^3) and deposited inside an electroelution chamber ("Little Blue Tank", ISCO) containing 1:10 dilution of native electrophoresis tank buffer. Anode and cathode chambers were filled with undiluted tank buffer and operated at 3 W at 10 ^{o}C for 3 h; 200 uL of electroeluted mayahuelin was recovered from the sample compartments.

Mayahuelin polyclonal antibody production

New Zealand white male rabbits (3 month-old) were used to raise antisera against mayahuelin by injecting native mayahuelin crosslinked to the acrylamide matrix from the containing gel slice. Acrylamide/antigen preparation was mixed with incomplete Freund's adjuvant (1:1 by volume) and injected subcutaneously at multiple sites in the rabbit's back. Following three injections over a period of 6 weeks, blood and sera were collected by standard procedures. A published protocol was followed for affinity purification of mayahuelin antibodies from the sera obtained (Madara et al., 1990).

Analysis by SDS/PAGE

The protein profile of each sample by SDS/PAGE was analyzed according to the Laemmli protocol (Laemmli, 1970). Samples in Laemmli sample buffer (plus 0.02% bromophenol blue) were loaded onto 4% stacking gels and resolved in 12% polyacrylamide SDS minigels (8 x 10 cm). Runs were performed using the Tris/glycine/SDS running buffer (24.8 mM/192 mM/1%) at 200 V/1h at 5 °C in a Mini-PROTEAN system (Bio-Rad). Gels were stained with a 0.1% Coomasie Brilliant Blue R-250 (CBB), 40% acetic acid, and 40% ethanol solution. For distaining, staining solution was replaced by distilled water and microwaved again during 4 min. Pink pre-stained protein ladder (15 -175 kDa [cat. MWP02, Nippon Genetics]) was employed as molecular weight marker.

Immunoblot analysis

Proteins separated by SDS/PAGE were electrophoretically transferred to nitrocellulose membranes (0.45 mM HATF, Millipore) using transfer buffer (25 mM Tris, 192 mM glycine, 20% isopropanol) in a Mini-PROTEAN (Bio-Rad) transfer system at 360 mA for 1h at 6 ^{o}C. Membranes were fixed (25% isopropanol, 10% acetic acid) for 1h with agitation. After distilled water wash, proteins on nitrocellulose were visualized with Ponceau S solution

(0.2% Ponceau S in 5% acetic acid). For western blot assay, membranes were blocked (1h at 25 ^{o}C) in a solution of 5% non-fat milk in TBS-T buffer (20mM Tris-HCl pH7.4, 150 mM NaCl, 0.05% Tween 20) and incubated with immunopurified anti-mayahuelin (1: 25 000) or HRP-goat-anti-rabbit IgG (H+L) (cat. 65-6120 from Zymed at 1:1000 dilution) as secondary antibody. All intermediate washes were done with TBS-T. Western blots were developed with SuperSignal West Femto (cat. 34095, Thermo Scientific) and exposed to X-Ray films (cat. 6040331, Kodak).

References:

- Chomczynski, P. and Sacchi, N. (1987). Single-step method of RNA isolation by acid guanidinium thiocyanate-phenol-chloroform extraction. *Anal. Biochem.* 162, 156-159.
- Doyle, J. J., and Doyle, J. L. (1987). A rapid DNA isolation procedure for small quantities of fresh leaf tissue. *Phytochemical Bulletin* 19: 11-15.
- Gietz, R. D., and Woods, R. (2002). Transformation of yeast by lithium acetate/single-stranded carrier DNA/polyethylene glycol Method. *Meth. Enzymol.* 350, 87-96.
- Heid, C. A., Stevens, J., Livak, K. J., and Williams, P. M. 1996. Real time quantitative PCR. *Genome Res.* 6, 986-994.
- Laemmli, U. K. (1970). Cleavage of structural proteins during the assembly of the head of bacteriophage T4. *Nature* 227, 680–685.
- Lledías, F., Hernández, F., Rivas, V., García-Mendoza, A., Cassab, G. I., and Nieto-Sotelo, J. (2017a). A rapid and reliable method for total protein extraction from succulent plants for proteomic analysis. *Protein J.* 36, 308–321. DOI 10.1007/s10930-017-9720-3.
- Lledías, F., Hernández, F., Rivas, V., García-Mendoza, A., Cassab, G. I., and Nieto-Sotelo, J. (2017b). Erratum to: A rapid and reliable method for total protein extraction from succulent plants for proteomic analysis. *Protein J.* 36: 523. DOI 10.1007/s10930-017-9739-5.
- Luján, R., Lledías, F., Martínez, L., Barreto, R., Cassab, G., and Nieto-Sotelo, J. (2009). Small heat-shock proteins and leaf cooling capacity account for the unusual heat tolerance of the central spike leaves in *Agave tequilana* var. Weber. *Plant Cell Environ*. 32, 1791-1803.

- Madara, P. J., Banghart, L. R., Jack, L. J. W., Neira, L. M., and Mather, I. H. (1990). Affinity purification of polyclonal antibodies from antigen immobilized *in situ* in sodium dodecyl sulfate-polyacrylamide gels. *Anal. Biochem.*187, 246-250.
- Martínez-Hernández, A., Mena-Espino, M. E., Herrera-Estrella, A. H., Martínez-Hernández P. (2010). Construcción de bibliotecas de ADNc y análisis de expresión génica por RT-PCR en agaves. *Rev. Latinoamer. Quím.* 38, 21-44.
- Ronquist, F., Teslenko, M., van der Mark, P. , Ayres, D., Darling, A., Höhna, S., et al. (2012). MrBayes 3.2: efficient Bayesian phylogenetic inference and model choice across a large model space. *Systematic Biol.* 61, 539-542.
- Simpson, J., Martínez-Hernández, A., Abraham Juárez, M. J., Delgado Sandoval, S., Sánchez Villarreal, A., and Cortés Romero, C. (2011). Genomic resources and transcriptome mining in *Agave tequilana*. *GCB Bioenergy.* 3, 25-36. doi: 10.1111/j.1757-1707.2010.01079.x
