# Supplementary material for: Mayahuelin, a Type I Ribosome Inactivating Protein: Characterization, Evolution, and Utilization in Phylogenetic Analyses of Agave
Source: Front Plant Sci. 2020 May 27;11:573. doi: 10.3389/fpls.2020.00573 (PMC7266874; doi:10.3389/fpls.2020.00573)
Supplement: Supplementary file 2 [file Data_Sheet_2.pdf]

Supplemental Figures and Tables

of manuscript:

Mayahuelin, a type I Ribosome Inactivating Protein: characterization, evolution, and utilization in phylogenetic analyses of *Agave*

submitted by:
Fernando Lledías, Jesús Gutiérrez, Aída Martínez-Hernández, Abisaí García-Mendoza, Eric Sosa, Felipe Hernández-Bermúdez, Tzvetanka D. Dinkova, Sandi Reyes, Gladys I. Cassab, and Jorge Nieto-Sotelo

to *Frontiers in Plant Science*

Supplemental Information (Figures)

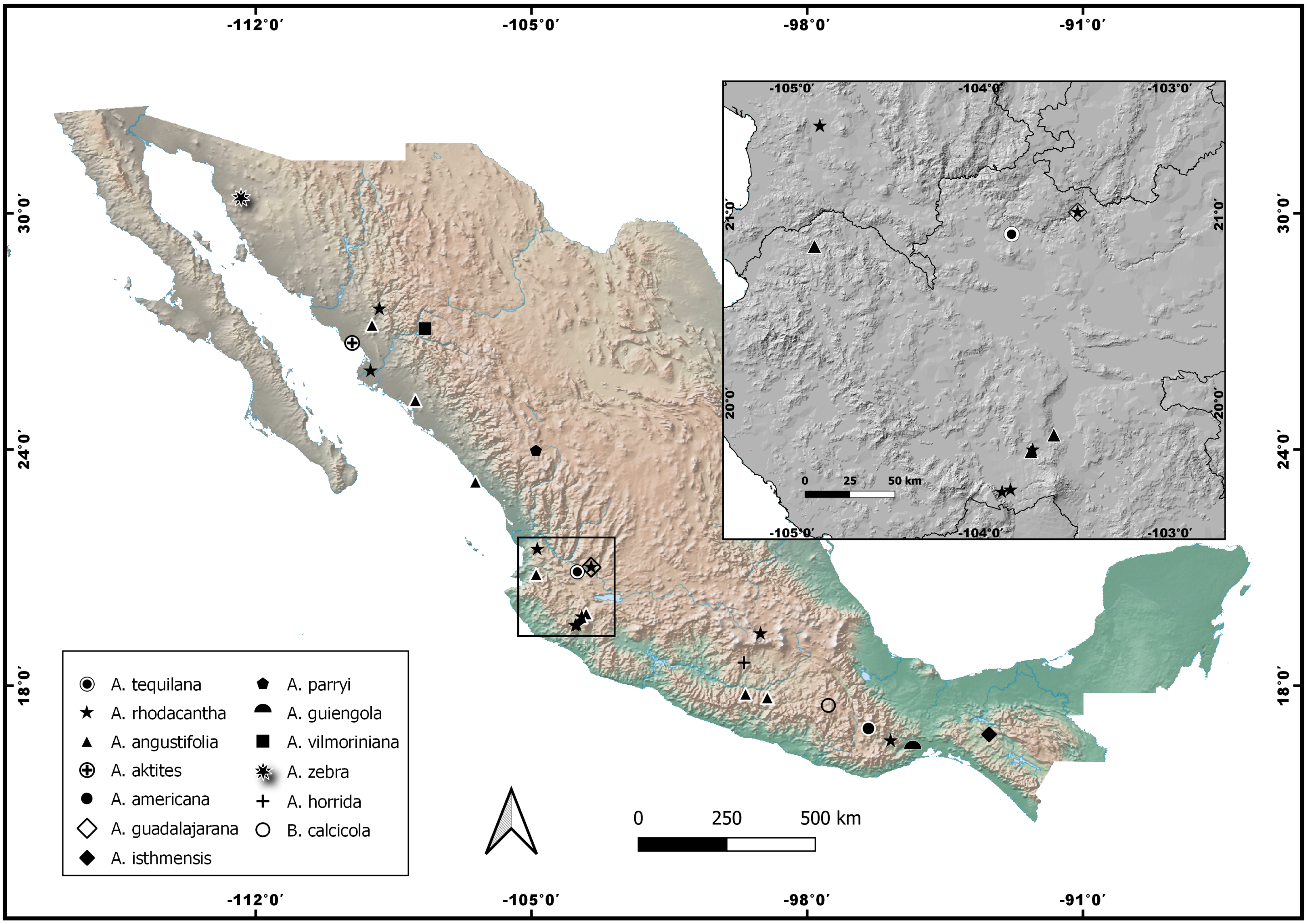
-112°0′
-105°0′
-98°0′
-91°0′
30°0′
24°0′
18°0′
-104°0′
-103°0′
21°0′
20°0′
0
25
50 km
250
500 km
A. tequilana
A. rhodacantha
A. angustifolia
A. aktites
A. americana
A. guadalajarana
A. isthmensis
A. parryi
A. guiengola
A. vilmoriniana
A. zebra
A. horrida
B. calcicola

Figure S1

```
aC3228_12      1 ----ASFKSSIFGILTLLLIFIAAAAADNCTSSEQQLKVKFEVNLNVPALTRKGYQNFLAELRKKLKVKDIGTT-NKVAV
aC3095_122     1 KKPWASFKSSIFGILMFVLIIIAAAAADNYTSSEQDLKVEFTDELDVPTLTRKGYQNFLANLRQKLKVKDIGTT-NKVAV
aC2764_16      1 KKPWASFKSSIFGILMFVLIIIAAAAADNYTSSEQDLKVIFTDELDVPTLTRKGYQNFLANLRQKLKVKDIGTT-NKVAV
aC3120_9       1 --TLASFKSSIFGILMFVLIIIAAAAADNYTSNEQDLKVIFTDELDVPTLTRKGYQNFLANLRQKLKVKDIGTT-NKVAV
aC1332_16      1 ---MASFKCSIFGILKLILIFI--AAADNYTSSEQHLKVKFEVNLDVGTLDPAGYRDFQAKLRDKLAARYIGPEGNNVAV
aC672_4        1 ---MASFKSNIFGILRLLLIIIAAAAADKYTSSEEHLKVKFEFSLDVQTLDSDEYIAFQANLRGKLKGGEIGPKGDAIAV
aC2795_15      1 ---MASFKSSIFGILTLLLIIIAATAADKYTSSEEHLKVKFELSLDVQTLDSDEYIAFQANLRGKLKGGEIGPKGDAIAV
aC279_3        1 ------FKSSIFGIWMFVLIII-AAAADNCTSSEQLLKVKFTDELDVQQLDSAGYIAFQANLREKLAERYIGTT-NKVAV
aC344_3        1 ---MASFKSSIFGILMFVLIIIAAAAADNCTSSEQLLKVKFTDELDVQQLDSDGYIAFQANLREKLAERYIGTT-NKVAV
aC643_5        1 ---MASFKCSIFGILMLILIIIAAAAADNHTNSEQLLKVKFELPLDVTTLDSNKYIAFQDKLRDELKGGEIGPQGNTVAV
aC630_3        1 -----SFKSSIFGILTLVLIFIAAAAADGYTSSEQHLKVKFEVNLDVRTLDAAGYRAFQDDLRKRLADKYIGPAGNNVAV
aC2762_3       1 KTQWASFKSSIFGMLTLILIFIAAAAADGYTSSEQHLKVKFEVNLDVRTLDAAGYRAFQADLRKRLADKYIGPAGNNVAV
consensus      1    ...**..***.....**.*...***..*..*. ***.*.. *.* .*.  .* .*...** .*. ..** .....**

aC3228_12     76 LPPDNKEDPEWFDLKLTGAGGEQTTVRFRVGSLYVVGYQMGT------------TWLELGNPNKGDTKHWIPGSQFLGFK
aC3095_122    80 LPPDNEKDPQWFDLKLTGADGAQTMVRFRVGNLYVVGYQMGT------------TWYEFG--KTGDTKHWITGSQFLGFR
aC2764_16     80 LPPDNEKDPQWFDLKLTGADGAQTTVRFRVGNLYVVGYQMGT------------TWYEFG--KTGDTKHWITGSQFLGFR
aC3120_9      78 LPPDNEKDPQWFDLKLTGADGAQTTVRFRVGNLYVVGYQMGT------------TWYEFG--KTGDTKHWITGSQFLGFR
aC1332_16     76 LPPDNEGAPQWFDLKLKGAGKA-ATVRFRVGNLYVVGYRQTPPDPLPTPPPPEPTWFELG--KTGD-RQWIPNSQFLGFK
aC672_4       78 LPPDNETDPQWFDLKLTGAGGANTTVRFRVGNLYVVGYLATTS----------STWFEFG--KKGD-KQWIPNSQFLGFK
aC2795_15     78 LPPDNETDPQWFDLKLTGAGGANTTVRFRVGNLYVVGYLATTS----------STWFEFG--KKGD-KQWIPNSQFLGFK
aC279_3       73 LPPDNEKNPEWFDLKLTGAGGAKTTVRFRVGNLYVVGYQMGT------------TWYEFGDPKKNDTKHWIDGSKFLGFR
aC344_3       77 LPPDNEKNPEWFDLKLTGAGGAKTTVRFRVGNLYVVGYQMGT------------TWLEFG--KTGDTKHWITGSQFLGFG
aC643_5       78 LPSDNKEDPEWFDVKLTGAGGAKTTVRFRVGNLYVVGYLATTP----------STWFEFG--KKGD-RQWIPNSQFLGFT
aC630_3       76 LPHDNEGAPQWFDLRLTGAGGAQTTVRFRVGNLDVVGYQMGT------------TWYEFG--KNGD-KQWIPNSQFLGFR
aC2762_3      81 LPPDNEGAPQWFDLRLTGAGGAQTTVRFRVGNLYVVGYQMGT------------TWYEFG--KKGD-KQWIPDSQFLGFR
consensus     81 ** **. .*.***..*.**......******.*.****....            **.*.*  ...*.. **..*.****.

aC3228_12    144 GDYVDLERTAKTAVTKINLNKNNWGEAVRTLATSVEADKR-AAALIVVVQLVSEACRFVILYNHISAKMNLPTPVYLEPW
aC3095_122   146 GDYGDLQRAAKMAVTNINLNKKNWGEAVKILATSEKAEER-AAALIVVIQLVSEACRFVTLSNHFSAKINSLTPLHVEEW
aC2764_16    146 GDYGDLQRAAKMAVTNINLNKKNWGEAVKILATSEKAEER-AAALIVVIQLVSEACRFVTLSNHFSAKINSLTPLHVEEW
aC3120_9     144 GDYGDLQRAAKMAVTNINLNKKNWGEAVKILATSEKAEER-AGALIVVIQLVSEACRFVTLSNHFSAKINSLTPLHVEEW
aC1332_16    152 DDYGALATAARKAVTDINLNRFNFETAVKTLATSTKGDESVAAALIVVAQLISEACRFVVLSNGLSEKLLSPS-VKLKEW
aC672_4      145 GDYVALERAAGKSLTEINLNRFNFEAAVKTLATSTKGDASVAAALIVVAQLISEACRFIVLSNGLSANLLSSQPVYLKEW
aC2795_15    145 GDYVALERAAGKSLTEINLNRFNFEAAVKTLATSTKGDTSVAAALIVVAQLISEACRFIVLSNGLSANLLSSQPVYLKEW
aC279_3      141 GDYGALTTAAKKKVTEINLNRYGFDAAVKTLATSTKSNER-AGALIVVAQLVSEACRFIVLSNGLSTKLLSSDPVYLKEW
aC344_3      143 GDYGELTTAAKKKVTEISLTKFKFEAAVKTLATSTKSNER-AEALIVVVQMVSEACRFVVLSNGLSTKLLSSDPVYLKEW
aC643_5      145 GDYVSLARAAGKKVTETNLNRYGFEAAVKTLATSTIGKER-AAALIVVAQLVSEACRFLILSNALSTKINDPAPLNLKQW
aC630_3      141 GDYGALANAAGKKVTEINLNVYGFEAAVKTLATSTKGNEG-AEALIVVAQLVSEACRFLILSNALSTRINDPTPLYLKQW
aC2762_3     146 GDYGALATAAGKKVTEINLNIYGFEAAVKTLATSTKGNER-AAALIVVAQLVSEACRFLILSNALSTRINDPAPLYLKEW
consensus    161 .**..* ..*.. .*...*.......**..****...... *.*****.*..*****..*.*..*..... .......*

aC3228_12    223 MLDDLERNWGKYSKILLCYNMFPTSYKFPELKINNEEIKAADDLRKKLGIVLNVQVDEKECKITGNDVELPSIIDFLAQV
aC3095_122   225 MLEDLEKNWGTYSKILLCYNMFPTSYKFPELKINNEVIRSADDLRKKLGIVLNVQVDKKECKITGTDVELPSIIDFLAQV
aC2764_16    225 MLEDLEKNWGTYSKILLCYNMFPTSYKFPELKINNEVIRSADDLRKKLGIVLNVQVDKKECKITGNDVELPSIIDFLAQV
aC3120_9     223 MLEDLEKNWGTYSKILLCYNMFPTSYKFPELKINNEVIKSADDLRKKLGIVLNVQVDKKECKITGTDVELPSIIDFLAQV
aC1332_16    231 MLEDLEKNWKTYSKILLCDDKFPDTYKFPKPTINQIVIATAADLRKKLGILLNVPVD-----------------------
aC672_4      225 MLNDLERKWGTYSEILFCYYTYPTSYKFPKQTINGKEVATAEKLRQKLGILLNVQVDTKVCKITANDVELPDVIAFQAQV
aC2795_15    225 MLNDLERKWGTYSEILFCYYTYPTSYNFPKQTINGKEVATAEKLRQKLGILLHVQVDKTKCKITANDVELP---------
aC279_3      220 MLVDLERKWGTYSEILLCYNQFPNSYKFPELTINQNVIPTADDLRKRLGILLNVQVDKTKCKITANDVELPDAIAFLAQV
aC344_3      222 MLVDLERKWGTYSEILLCYNQFPNSYKFPELTINQNVIPD----------------------------------------
aC643_5      224 MLNDLEREWGTYSEILLCYNQFPTTYNFPKLTINQNVITTADELRKKLGILLNVQVDTKVCKITANDVELPDVIAFLAKV
aC630_3      220 MLDDLEREWGTYSEILMCYNNFPGTYNFPKPIINQNVIATANELRKILGILLNVEVNYQVCKITANDVELPDVIAFLAKV
aC2762_3     225 MLNDLQREWGTYSEILLCDNQFPTTYNFPKPIINQNVIATADELRKILGILLNVQVNNKVCKITANDVELPDV-------
consensus    241 **.**.. *..**.**.*.. .*..*.** ..**. .. .................. . ........... .. .....

aC3228_12    303 QNPVALAGVEA
aC3095_122   305 QNPVAVAGVEA
aC2764_16    305 QNPVAVPGVEA
aC3120_9     303 QNPVAVAGVEA
aC1332_16        -----------
aC672_4      305 QNPVELAGVEA
aC2795_15        -----------
aC279_3      300 QNAVELAGVEA
aC344_3          -----------
aC643_5      304 QNAVELVGVEA
aC630_3          QNPVELAGVEA
aC2762_3         -----------
consensus    321 .. . . ....
```

Figure S2

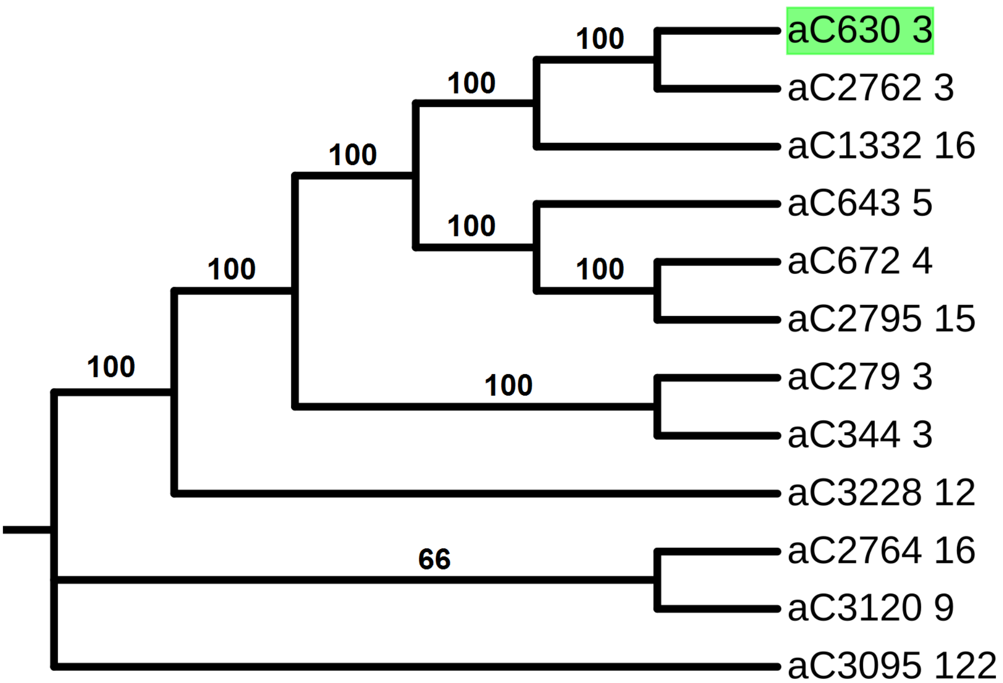
100
100
100
100
100
100
100
100
66
aC630 3
aC2762 3
aC1332 16
aC643 5
aC672 4
aC2795 15
aC279 3
aC344 3
aC3228 12
aC2764 16
aC3120 9
aC3095 122

Figure S3

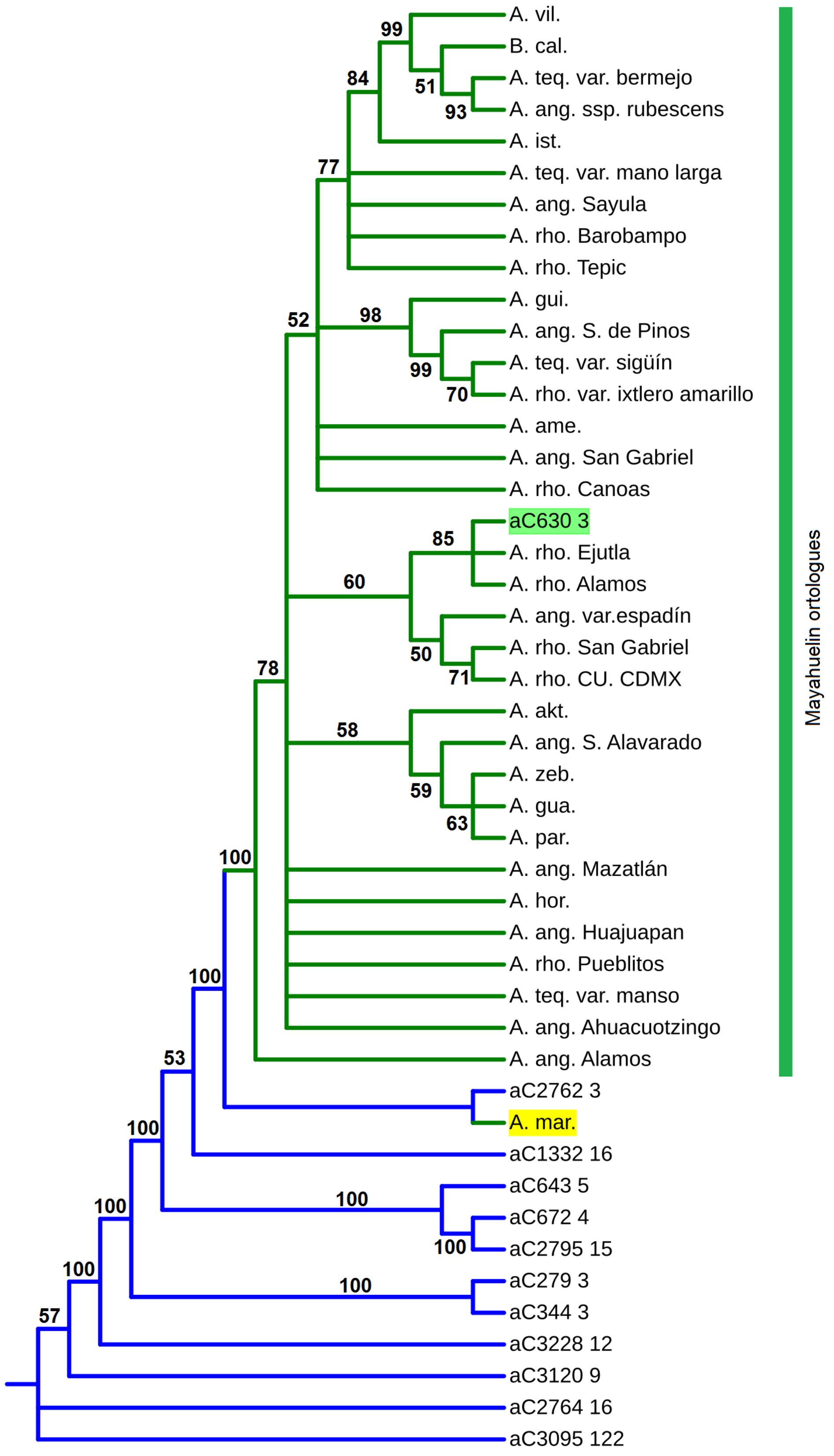

A. vil.
B. cal.
A. teq. var. bermejo
A. ang. ssp. rubescens
A. ist.
A. teq. var. mano larga
A. ang. Sayula
A. rho. Barobampo
A. rho. Tepic
A. gui.
A. ang. S. de Pinos
A. teq. var. sigüín
A. rho. var. ixtlero amarillo
A. ame.
A. ang. San Gabriel
A. rho. Canoas
aC630 3
A. rho. Ejutla
A. rho. Alamos
A. ang. var.espadín
A. rho. San Gabriel
A. rho. CU. CDMX
A. akt.
A. ang. S. Alavarado
A. zeb.
A. gua.
A. par.
A. ang. Mazatlán
A. hor.
A. ang. Huajuapan
A. rho. Pueblitos
A. teq. var. manso
A. ang. Ahuacuotzingo
A. ang. Alamos
aC2762 3
A. mar.
aC1332 16
aC643 5
aC672 4
aC2795 15
aC279 3
aC344 3
aC3228 12
aC3120 9
aC2764 16
aC3095 122
99
84
51
93
77
98
52
99
70
85
60
50
71
78
58
59
63
100
100
53
100
100
100
100
100
100
57
Mayahuelin ortologues

Figure S4

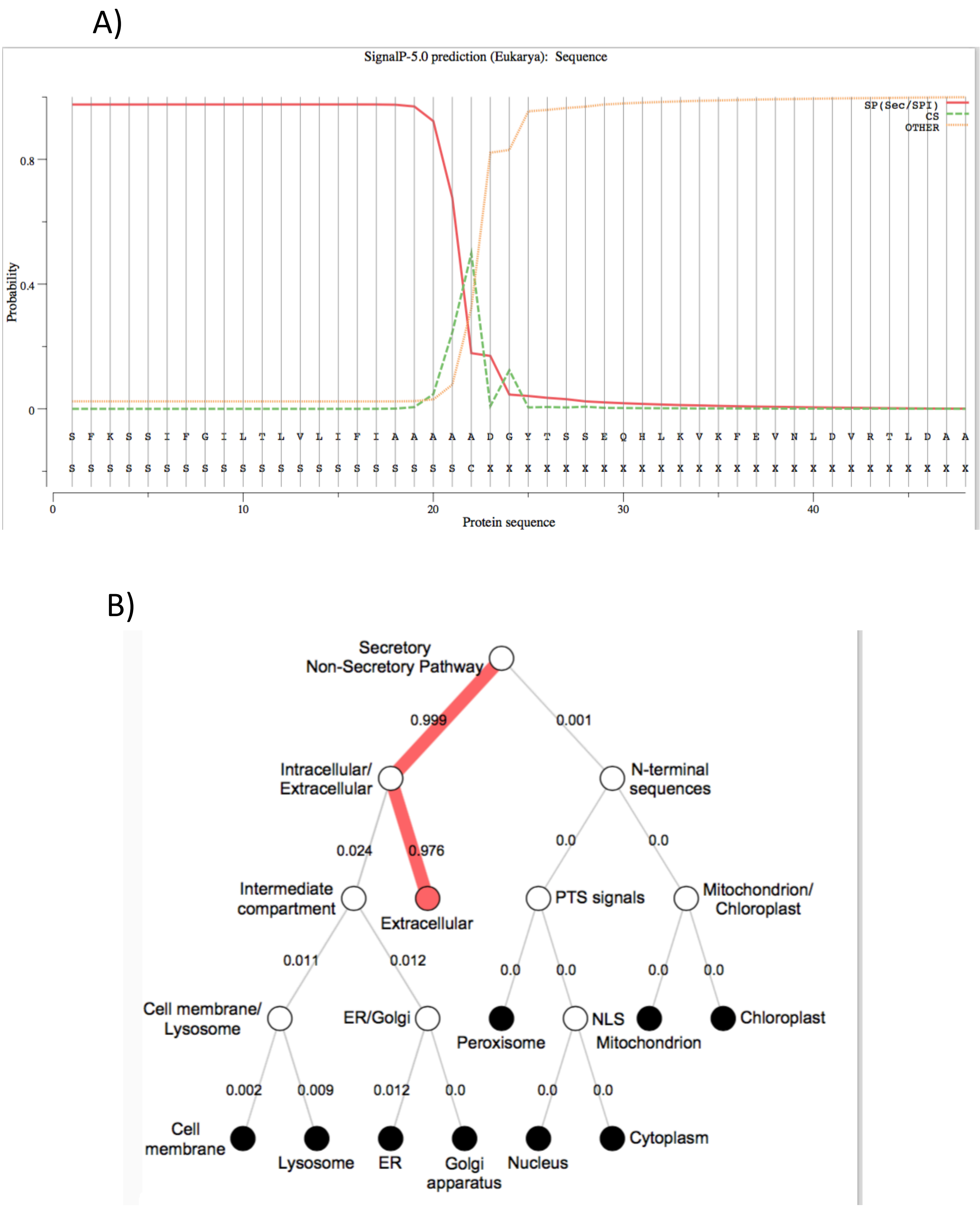
A)
SignalP-5.0 prediction (Eukarya): Sequence
SP(Sec/SPI)
CS
OTHER
Probability
0.8
0.4
0
S F K S S I F G I L T L V L I F I A A A A A D G Y T S S E Q H L K V K F E V N L D V R T L D A A
S S S S S S S S S S S S S S S S S S S S S C X X X X X X X X X X X X X X X X X X X X X X X X X X
0
10
20
30
40
Protein sequence
B)
Secretory
Non-Secretory Pathway
0.999
0.001
Intracellular/
Extracellular
N-terminal
sequences
0.024
0.976
0.0
0.0
Intermediate
compartment
Extracellular
PTS signals
Mitochondrion/
Chloroplast
0.011
0.012
0.0
0.0
0.0
0.0
Cell membrane/
Lysosome
ER/Golgi
Peroxisome
NLS
Mitochondrion
Chloroplast
0.002
0.009
0.012
0.0
0.0
0.0
Cell
membrane
Lysosome
ER
Golgi
apparatus
Nucleus
Cytoplasm

Figure S5

```
Mayahuelin       1 -------VKFEVNLDVRTLDAAGYRAFQDDLRKRLADKYIGPAGNNVAVLPHDN--EGAP
Charybdin        1 SQCKAMTVKFTVELDIERLTGQTYTDFIKNLRRSLATWYL----HGVPVLPLYN-QEADP
Ricin_chainA     1 IFPK---QYPIINFTTAGATVQSYTNFIRAVRGRLTTGADVR--HEIPVLPNRVGLPINQ
Abrin_chainA     1 E-------DRPIKFSTEGATSQSYKQFIEALRERLRGG--LI--HDIPVLPDPTTLQERN
Saporin          1 -------VT-SITLDLVNPTAGQYSSFVDKIRNNVKDPNLKYGGTDIAVIG----PPSKE
PAP              1 I-------N-TITFDAGNATINKYATFMESLRNEAKDPSLKC--YGIPMLPN---TNSTI
Trichosanthin    1 -------VEGDVSFRLSGATSSSYGVFISNLRKALPNER-KL--YDIPLLRS--SLPGSQ
consensus        1        .   . ...  .. ..*  *.. .*  .    .     ......

Mayahuelin      52 QWFDLRLTGAGGAQTTVRFRVGNLDVVGYQMGTTW-----YEFG-KN-GDK-------QW
Charybdin       56 RGFDLKLTFR-GQVTTVRIHRDDLVLRGYQMQGAG----KWLEL-ER-PST-Q--T-GHL
Ricin_chainA    56 RFILVELSNHAELSVTLALDVTNAYVVGYRAGNS-----AYFFHPDNQEDAEA--I-THL
Abrin_chainA    50 RYITVELSNSDTESIEVGIDVTNAYVVAYRAGTQ-----SYFLR-DAP--SSA--S-DYL
Saporin         49 KFLRINFQSS-RGTVSLGLKRDNLYVVAYLAMDNTNVNRAYYFKSEIT-SAE---L-TAL
PAP             48 KYLLVKLQGASLKTITLMLRRNNLYVMGYSDPYDN--KCRYHIFNDIK-GTEYSDVENTL
Trichosanthin   49 RYALIHLTNYADETISVAIDVTNVYIMGYRAGDT-----SYFFN-EAS-ATEA--A-KYV
consensus       61 ... ....     ........ ......* ..        ...  .    ..       .

Mayahuelin      98 I-----PNSQFLGFRGDYGALANAAG-----KKVTEINLNVYGFEAAVKTLAT---S-T-
Charybdin      105 I-----EGSELLEFGPSYEELAAAAQ-----QDILDISYNKNALQDAVSKLAV---S-T-
Ricin_chainA   108 FTD--VQNRYTFAFGGNYDRLEQLAG-----NLRENIELGNGPLEEAISALYYYSTGGT-
Abrin_chainA    99 FTG---TDQHSLPFYGTYGDLERWAH-----QSRQQIPLGLQALTHGISFFR---SGGN-
Saporin        103 FPEATTANQKALEYTEDYQSIEKNAQITQGDKSRKELGLGIDLLLTFMEAVN---KKARV
PAP            105 CPSSNPRVAKPINYNGLYPTLEKKAGV----TSRNEVQLGIQILSSDIGKIS---GQGSF
Trichosanthin   99 FKD--AMRKVTLPYSGNYERLQTAAG-----KIRENIPLGLPALDSAITTLFY---Y---
consensus      121 .          . . . *  ..   *.       . .. ... ..  ... .      ..

Mayahuelin     143 KGNEGAEALIVVAQLVSEACRFLILSNALSTRINDPTPLYLKQWMLDDLEREWGTYSEIL
Charybdin      150 NTRDRARSLIVVSQMFCEATRFVDIANHFAFNLESSEPVKLPQWMQNDLEKNWVRFSFMI
Ricin_chainA   160 QLPTLARSFIICIQMISEAARFQYIEGEMRTRIRYNRR-SAPDPSVITLENSWGRLSTAI
Abrin_chainA   147 DNEEKARTLIVIIQMVAEAARFRYISNRVRVSIQTGTA-FQPDAAMISLENNWDNLSRGV
Saporin        160 -VKNEARFLLIAIQMTAEVARFRYIQNLVTKNFP--NK-FDSDNKVIQFEVSWRKISTAI
PAP            158 TEKIEAKFLLVAIQMVSEAARFKYIENQVKTNFN--RD-FSPNDKVLDLEENWGKISTAI
Trichosanthin  146 NANSAASALMVLIQSTSEAARYKFIEQQIGKRVD--KT-FLPSLAIISLENSWSALSKQI
consensus      181      *. .....*...*..*.... . .   .      . .   .. .*  * ..* ..

Mayahuelin     203 MCYNNFPGTYNFPKPII--NQN--VIATANE--LRKILGILLN-----------------
Charybdin      210 LKSNA-DPCYKFEPQTI--YGK--IIKTADE--LLNFLGIVEQHPDT----------R--
Ricin_chainA   219 QE--SNQGAFASPIQLQRRNGSKFSVYDVSI--LIPIIALMVYRCAP-------------
Abrin_chainA   206 QE--SVQDTFPNQVTLTNIRNEPVIVDSLSHP-TVAVLALMLFVCNP-------------
Saporin        216 YGD-AKNGVFNKDYDFG--FGKVRQVKD-----L--QMGLLMYLG---------------
PAP            215 HNS--KNGALPKPLELKNADGTKWIVLRVDE--IKPDVGLLNYVNG--------------
Trichosanthin  203 QIASTNNGQFESPVVLINAQNQRVTITNVDAGVVTSNIALLLNRNNMAAMDDDVPMTQSF
consensus      241       .. .  .. .    .   ..  .    .   ......

Mayahuelin     240 -VEVNYQ
Charybdin      251 SPPCAAG
Ricin_chainA   262 -PPSSQF
Abrin_chainA   250 -----PN
Saporin        251 ----KPK
PAP            257 --TCQAT
Trichosanthin  263 GCGSYAI
consensus      301
```

Figure S6

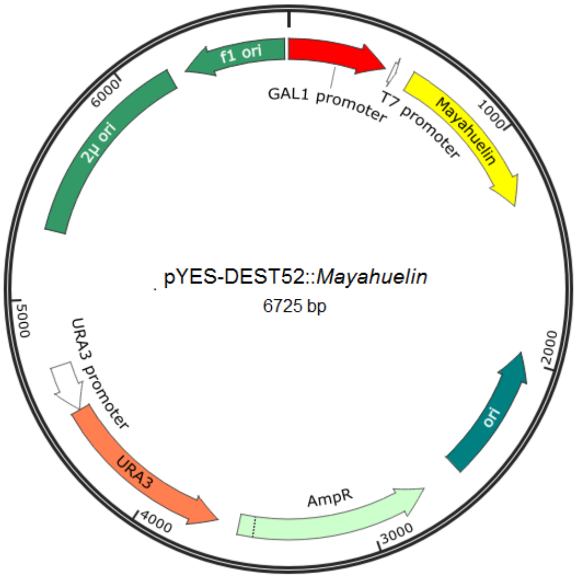

f1 ori
GAL1 promoter
T7 promoter
Mayahuelin
6000
1000
2μ ori
pYES-DEST52::*Mayahuelin*
6725 bp
5000
URA3 promoter
2000
ori
URA3
AmpR
4000
3000

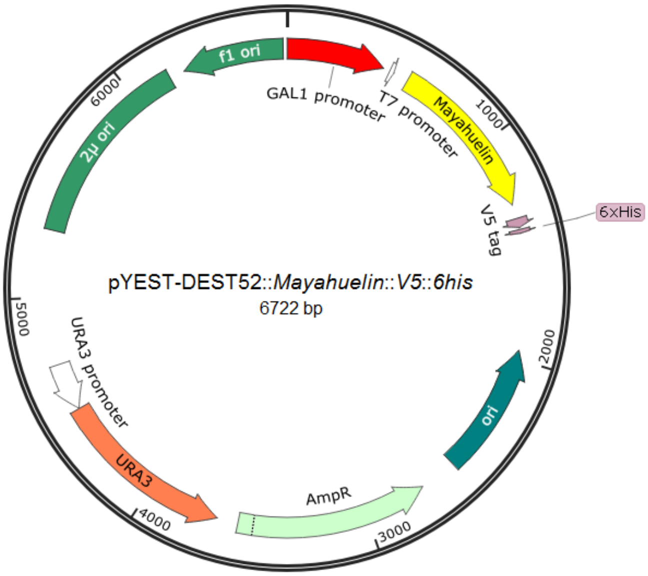

f1 ori
GAL1 promoter
T7 promoter
Mayahuelin
6000
1000
2μ ori
V5 tag
6xHis
pYEST-DEST52::*Mayahuelin*::*V5*::*6his*
6722 bp
5000
URA3 promoter
2000
ori
URA3
AmpR
4000
3000

Figure S7

CTTCTTTCAAGTCCAGTATCTTCGGAATATTGACGTTAGTTCTGATCTTCATTGCTGCAG

S F K S S I F G I L T L V L I F I A A A

CTGCTGCTGACGGCTACACTAGCAGCGAGCAACATTTGAAGGTGAAATTTGAGGTCAACC

A A D G Y T S S E Q H L K V K F E V N L

TCGATGTACGAACGCTAGACGCTGCAGGTTACAGAGCCTTCCAGGACGATCTCCGCAAAA

D V R T L D A A G Y R A F Q D D L R K R

GGTTGGCAGACAAGTACATAGGACCTGCAGGCAACAATGTTGCGGTGCTGCCCCACGACA

L A D K Y I G P A G N N V A V L P H D N

ACGAAGGAGCCCCGCAATGGTTCGACCTGAGACTAACAGGCGCCGGAGGAGCACAGACCA

E G A P Q W F D L R L T G A G G A Q T T

CAGTGAGGTTTCGCGTCGGCAACCTCGACGTGGTCGGTTATCAGATGGGGACGACCTGGT

V R F R V G N L D V V G Y Q M G T T W Y

ACGAGTTCGGGAAAAACGGCGACAAGCAATGGATTCCCAACTCTCAGTTCTTGGGCTTCA

E F G K N G D K Q W I P N S Q F L G F R

GAGGCGACTACGGGGCACTGGCAAACGCAGCAGGCAAGAAAGTGACGGAGATAAACCTTA

G D Y G A L A N A A G K K V T E I N L N

ATGTATACGGTTTCGAAGCAGCTGTGAAAACACTCGCCACGTCCACAAAAGGCAACGAGG

V Y G F E A A V K T L A T S T K G N E G

GGGCAGAGGCACTGATAGTCGTGGCTCAGTTGGTCTCCGAAGCCTGCAGATTCCTCATCC

A E A L I V V A Q L V S E A C R F L I L

TCTCCAACGCTCTCTCAACCAGGATAAACGACCCAACGCCTCTCTATCTCAAGCAATGGA

S N A L S T R I N D P T P L Y L K Q W M

TGCTGGATGATCTAGAGAGGGAATGGGGGACGTACTCTGAGATTTTGATGTGCTACAATA

L D D L E R E W G T Y S E I L M C Y N N

ACTTTCCCGGCACTTACAACTTCCCAAAACCGATCATAAACCAAAATGTAATCGCGACGG

F P G T Y N F P K P I I N Q N V I A T A

CTAACGAACTGCGCAAAATACTTGGTATCCTGCTCAACGTTGAAGTTAACTACCAAGTGT

N E L R K I L G I L L N V E V N Y Q V C

GCAAAATCACTGCGAATGATGTTGAGCTGCCAGATGTTATTGCTTTTCTAGCAAAAGTGC

K I T A N D V E L P D V I A F L A K V Q

AGAATCCTGTCGAGCTCGCGGGAGTTGAAGCTTGAACTCCGTCATCTCGTCCGCTGTAGC

N P V E L A G V E A *

TGCTGGTGGTGTTTGTTATATATCTACTTGGGCTTGTTATAATAGTAATAAAACAACCGG

CAGTTTGTATCGTTGATGTTTATATACTGGTTCTCTGTGTACGGAACTCCTAAGTATTAA

TAATAATATTTCATGTTGTGTTACAAAAAAAAAAAAA

Figure S8

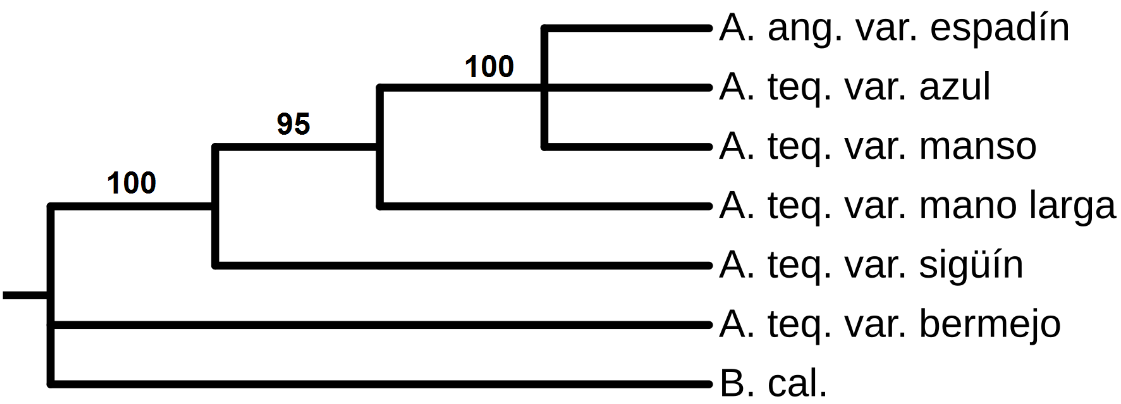
100
95
100
A. ang. var. espadín
A. teq. var. azul
A. teq. var. manso
A. teq. var. mano larga
A. teq. var. sigüín
A. teq. var. bermejo
B. cal.

Figure S9

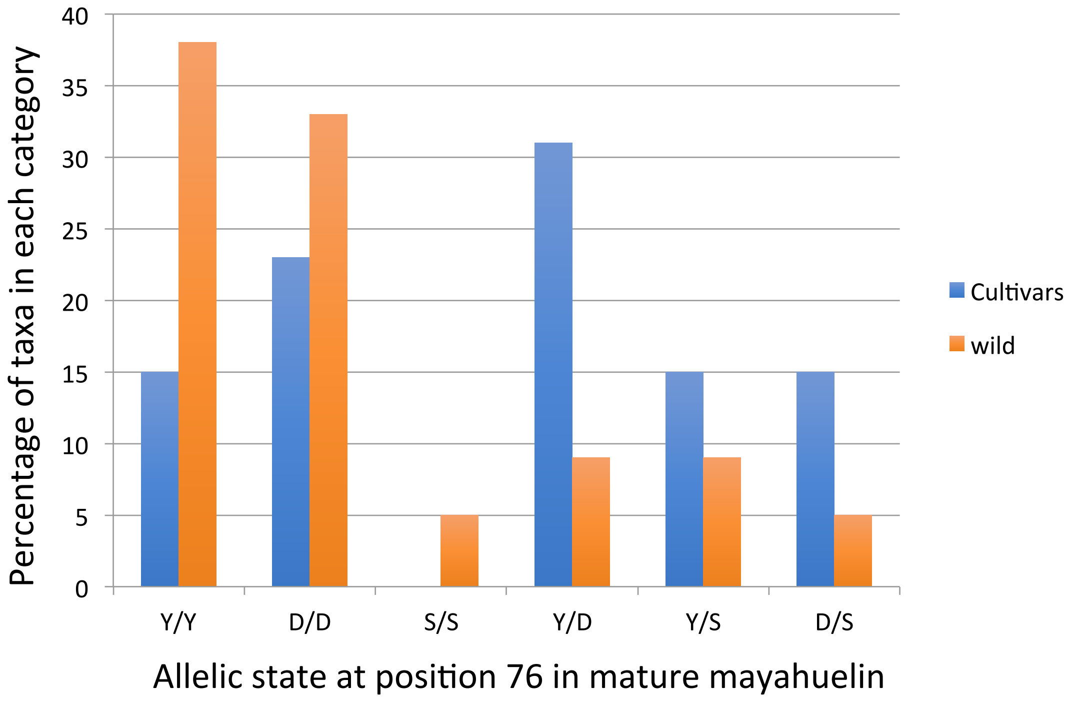
Percentage of taxa in each category
40
35
30
25
20
15
10
5
0
Y/Y
D/D
S/S
Y/D
Y/S
D/S
Allelic state at position 76 in mature mayahuelin
Cultivars
wild

Figure S10

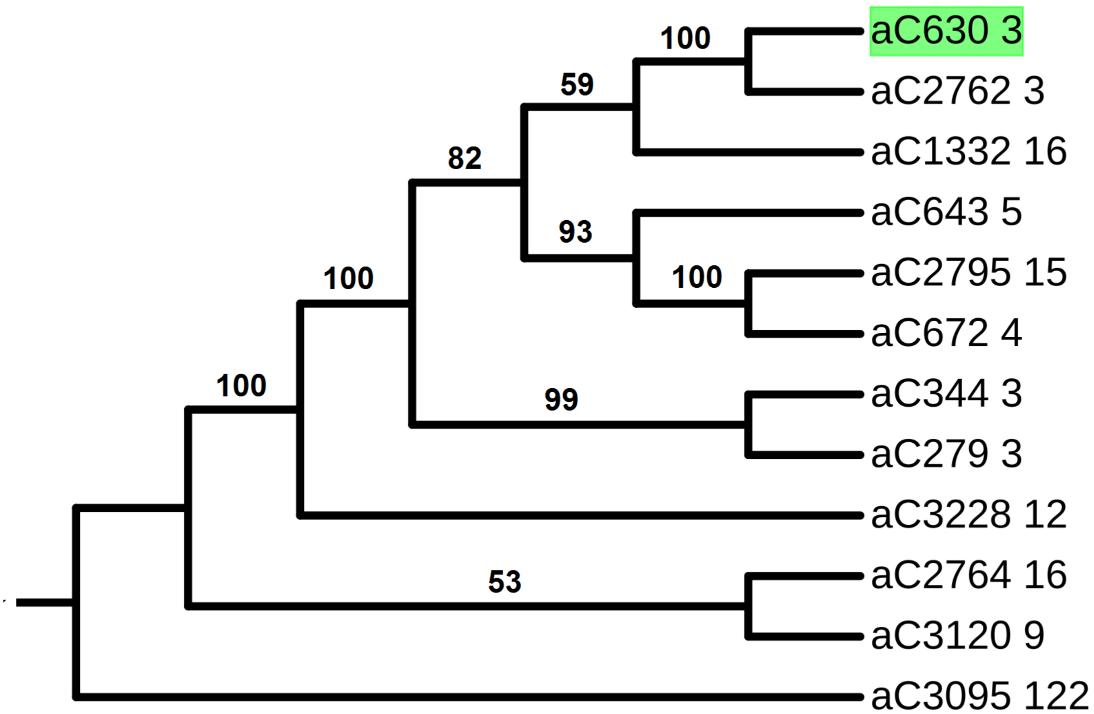
100
59
82
93
100
100
100
99
53
aC630 3
aC2762 3
aC1332 16
aC643 5
aC2795 15
aC672 4
aC344 3
aC279 3
aC3228 12
aC2764 16
aC3120 9
aC3095 122

Figure S11

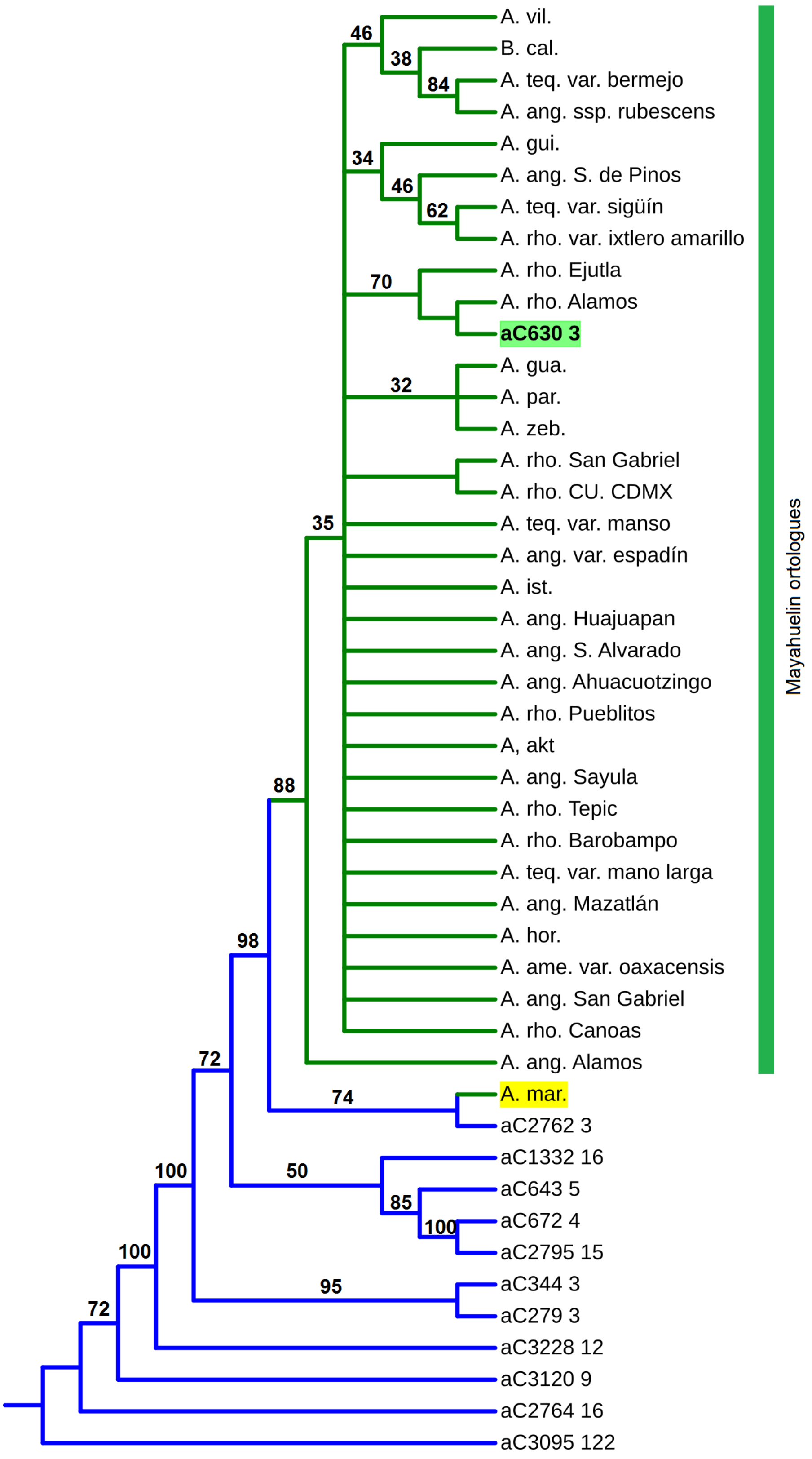
46
38
84
34
46
62
70
32
35
88
98
72
100
100
72
74
50
85
100
95
A. vil.
B. cal.
A. teq. var. bermejo
A. ang. ssp. rubescens
A. gui.
A. ang. S. de Pinos
A. teq. var. sigüín
A. rho. var. ixtlero amarillo
A. rho. Ejutla
A. rho. Alamos
aC630 3
A. gua.
A. par.
A. zeb.
A. rho. San Gabriel
A. rho. CU. CDMX
A. teq. var. manso
A. ang. var. espadín
A. ist.
A. ang. Huajuapan
A. ang. S. Alvarado
A. ang. Ahuacuotzingo
A. rho. Pueblitos
A, akt
A. ang. Sayula
A. rho. Tepic
A. rho. Barobampo
A. teq. var. mano larga
A. ang. Mazatlán
A. hor.
A. ame. var. oaxacensis
A. ang. San Gabriel
A. rho. Canoas
A. ang. Alamos
A. mar.
aC2762 3
aC1332 16
aC643 5
aC672 4
aC2795 15
aC344 3
aC279 3
aC3228 12
aC3120 9
aC2764 16
aC3095 122
Mayahuelin ortologues

Figure S12

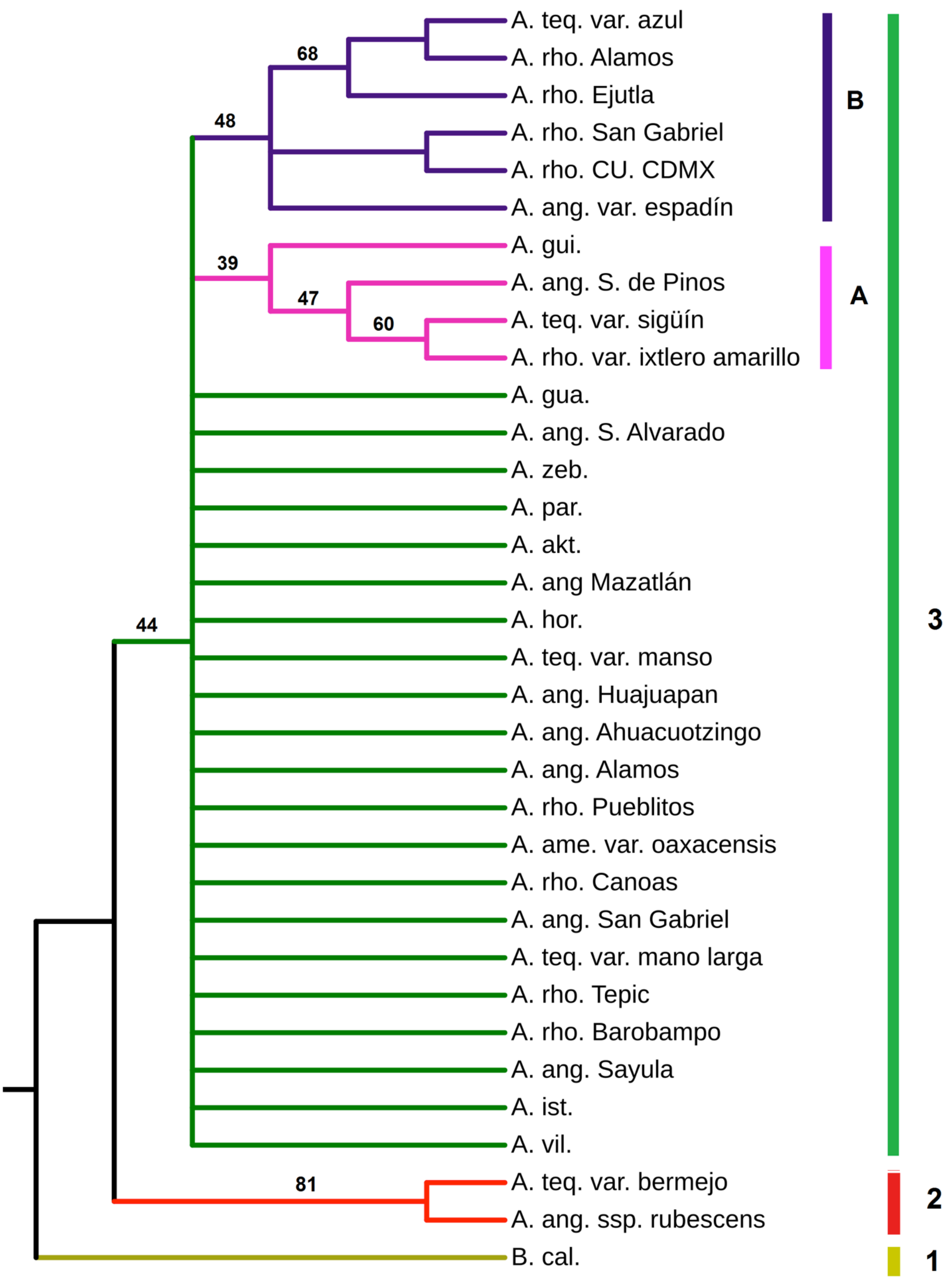
68
48
39
47
60
44
81
A. teq. var. azul
A. rho. Alamos
A. rho. Ejutla
A. rho. San Gabriel
A. rho. CU. CDMX
A. ang. var. espadín
A. gui.
A. ang. S. de Pinos
A. teq. var. sigüín
A. rho. var. ixtlero amarillo
A. gua.
A. ang. S. Alvarado
A. zeb.
A. par.
A. akt.
A. ang Mazatlán
A. hor.
A. teq. var. manso
A. ang. Huajuapan
A. ang. Ahuacuotzingo
A. ang. Alamos
A. rho. Pueblitos
A. ame. var. oaxacensis
A. rho. Canoas
A. ang. San Gabriel
A. teq. var. mano larga
A. rho. Tepic
A. rho. Barobampo
A. ang. Sayula
A. ist.
A. vil.
A. teq. var. bermejo
A. ang. ssp. rubescens
B. cal.
B
A
3
2
1

Figure S13

Supplemental Information (Tables)

Table S1. List of species and provenance of specimens studied.

Species name	Condition	Specimen collection number	Locality	Location	Geographical coordinates	Altitude (m.a.s.l.)
A. tequilana var. *azul*	Cultivar	N.A.	Tequila, Jal.	Jardín Botánico Casa Sauza	20° 53.677' N, 103° 50.281' W	1173
A. tequilana var. *mano larga*	Cultivar	JNS24	Tequila, Jal.	Jardín Botánico Casa Sauza	20° 53.677' N, 103° 50.281' W	1173
A. tequilana var. *manso*	Cultivar	JNS27	Tequila, Jal.	Jardín Botánico Casa Sauza	20° 53.677' N, 103° 50.281' W	1173
A. tequilana var. *sigüín*	Cultivar	JNS28	Tequila, Jal.	Jardín Botánico Casa Sauza	20° 53.677' N, 103° 50.281' W	1173
A. tequilana var. *bermejo*	Cultivar	JNS29	Tequila, Jal.	Jardín Botánico Casa Sauza	20° 53.677' N, 103° 50.281' W	1173
A.angustifolia var. *espadín*	Cultivar	JNS63	San Dionisio Ocotepec, Oax.	Donated by a local mescal producer	16° 47.608' N, 96° 23.193' W	1731
A. rhodacantha	Cultivar	JNS11	Tepic, Nay.	Highway to Nogales	21° 28.032' N, 104° 51.213' W	923
A. rhodacantha	Wild	JNS40	San Gabriel, Jal.	Sayula-San Gabriel road, Km 31.5	19° 45.070' N, 103° 43.591' W	1652
A. rhodacantha var. *ixtlero amarillo*	Cultivar	JNS46	Zapotitlán de Vadillo, Jal.	2 Km SW of town	19° 32.375' N, 103° 50.641' W	964
A. rhodacantha	Wild	JNS48	Canoas, Jal.	Edge of Armería river	19° 31.685' N, 103° 53.334' W	677
A. rhodacantha	Wild	JNS54	Pueblitos, Jal.	1 km SE of town	21° 00.668' N, 103° 29.305' W	1279

A. rhodacantha	Wild	AG9173	Alamos, Son.	San Bernardo-La Mesa Colorada road, Km 14	27° 34.178’ N, 108° 51.66’ W	226
A. rhodacantha	Cultivar	JE06	UNAM, CdMx	Metrobus 'CU' station, next to bridge, E side of avenue	19° 19.435' N, 99° 11.33' W	2298
A. rhodacantha	Cultivar	AG10959	Ejutla, Oax.	1 km E of El Vado	16° 36.193’ N, 96° 53.128’ W	1522
A. rhodacantha	Wild	DS1686	Sierra Barobampo, Sin.	Mayo-Yoreme territory	25° 59.772' N, 109° 5.234' W	279
A. angustifolia	Wild	JNS37	Sayula, Jal.	Sayula-San Gabriel road, Km 5.6	19° 49.929' N, 103° 36.851' W	1609
A. angustifolia	Wild	JNS43	San Gabriel, Jal.	Sayula-San Gabriel road, Km 33 and 0.5 Km above vista point	19° 44.636' N, 103° 44.137' W	1577
A. angustifolia	Wild	JNS05	Santiago de Pinos, Jal.	11.6 Km N of intersection San Sebastián-Santiago de Pinos	20° 49.834’ N, 104° 52.911’ W	844
A. angustifolia	Cultivar	MRL87	Huajuapan, Oax.	N.A.	N.A.	N.A.
A. angustifolia	Cultivar	ES01	Ahuacuotzingo, Gro.	Trapiche Viejo	17° 42.06' N, 99° 00.933' W	1295
A. angustifolia	Wild	DS1663	Mazatlán, Sin.	Near lighthouse, west flank of Mirador de Cristal	23° 10.657' N, 106° 25.525' W	20
A. angustifolia	Wild	DS1664	Salvador Alvarado, Sin.	7 km south of Caitime	25° 14.953' N, 107° 56.963' W	89
A. angustifolia	Wild	DS1682	Alamos, Sonora	Near Piedras Verdes mine	27° 9.670' N, 109° 2.926' W	256
A. angustifolia ssp. *rubescens*	Wild	SC04	Milpillas, Gro.	Edge of Iguala-Chilpancingo road	17° 47.614' N, 99° 34.093' W	713

A. americana var. *oaxacensis*	Cultivar	AG10531	Tlacolula, Oax.	2 km S of Tanivet	16° 54.878’ N, 96° 26.875’ W	1650
A. guadalajarana	Wild	JNS58	Pueblitos, Jal.	1 km SW of town in maize field	21° 00.668' N, 103° 29.305' W	1279
A. guiengola	Wild	AG5685	Santo Domingo Tehuantepec, Oax.	*Cerro Guiengola* archaeological site	16° 23.198’ N, 95° 19.400’ W	420
A. horrida	Wild	SC06	Taxco de Alarcón Gro.	Near shooting grounds	18° 35.302' N, 99° 36.377' W	2284
A. isthmensis	Wild	AG4177	Ocozocuautla, Chis.	1 km N of Ocozocuautla	16° 47.479’ N, 93° 23.239’ W	867
A. parryi	Wild	AG4968	La Palmita, Dgo.	35 km W of Durango near Durango-Mazatlán road	23° 57.886’ N, 104° 53.263’ W	2220
A. vilmoriniana	Wild	6000	Batopilas, Chih.	Santiago creek, 6 km N of Batopilas	27° 4.215’ N, 107° 42.230’ W	675
A. zebra	Wild	AG8876	Caborca, Son.	NW side of sierra El Viejo	30° 23.536’ N, 112° 22.455’ W	698
A. aktites	Wild	DS1679	Yavaros, Son.	2 km south of Yavaros lighthhouse	26° 42.408' N, 109° 33.135' W	10
Beschorneria calcicola	Wild	AG8771	Teposcolula, Oax.	Cerro Viejo	17° 30.07' N, 97° 27.955' W	2352

Table S2. List of mayahuelin peptide fragments and their amino acid sequences obtained *de novo* by nano-electrospray LC-MS/MS. Experiment number 2.

No.	Sequence	Modifi-cations	Pre-cursor ion	Molecular weight MH^+ (Da)	Charge
1	VKFEVNLDVR	none	406.9	1218.68	3
2	TLDAAGYR	none	433.72	866.43	2
3	AFQDDLR	none	432.71	864.42	2
4	AFQDDLRK	none	496.76	992.51	2
5	YIGPAGNNVAVLPHDNEGAPQWFDLR	none	950.8	2850.39	3
6	LTGAGGAQTTVR	Oxi-dation (+16)	566.31	1131.61	2
7	FRVGNLDVVGYQmGTTWYEFGK	none	861.75	2583.23	3
8	QWIPNSQFLGFR	none	746.89	1492.77	2
9	VTEINLNVYGFEAAVK	none	883.97	1766.93	2
10	AEALIVVAQLVSEACR	none	864.97	1728.93	2
11	FLILSNALSTR	none	617.86	1234.71	2
12	QWMLDDLER	none	603.28	1205.56	2
13	PIINQNVIATANELRK	none	598.67	1794.02	3
14	ILGILLNVEVNYK	none	744.45	1487.9	2

Table S3. Nucleotide sequence of primers used in this work.

Name	Nucleotide sequence	Application
**F1**	5' CACCTAAATAATGTCTGTGAAATTTGAGGTCAACCT 3'	Cloning
**R2**	5' TTGGTAGTTAACTTCAACGTT 3'	
**R1**	5' TCATTGGTAGTTAACTTCAACGTT 3'	
**T7**	5′ TAATACGACTCACTATAGGG 3′	Insert verification
**R-pYESDest52**	5′ CTCCTTCCTTTTCGGTTAGA 3′	
**F2**	5' GTGAAATTTGAGGTCAACCT 3'	Phylogenetic analyses
**R2**	5' TTGGTAGTTAACTTCAACGTT 3'	
**F6**	5' TCAACCTCGATGTACGAA C 3'	
**R6**	5' GGTAGTTAACTTCAACGTTGA 3'	
**Fwd3_630**	5' GCTGCCCCACGACAACGAAGGAG 3'	Quantitative PCR
**Rev3_630**	5' AAGTGCCGGGAAAGTTATTGTAGC 3'	

Table S4. List of ESTs from *Agave tequilana* var. *azul* encoding RIP family members.

**Name**	**Genbank accession number**
aC2795_15	MT015979
aC672_4	MT015975
aC643_5	MT015976
aC1332_16	MT015980
aC2762_3	MT015974
aC630_3	MN913554
aC344_3	MT015972
aC279_3	MT015971
aC3228_12	MT015978
aC3120_9	MT015977
aC3095_122	MT015982
aC2764_16	MT015981

Table S5. Amino acid and codon found at position 76 in mayahuelin proteins/genes from different Agavoideae species. Mayahuelin Genbank accession numbers for each taxa are found in Table S6.

**Taxa**	**Location of origin**	**aa residue at position 76**
A. tequilana var. *azul*	Tequila, Jal.	D/D
A. tequilana var. *mano larga*	Tequila, Jal.	Y/Y
A. tequilana var. *manso*	Tequila, Jal.	Y/D
A. tequilana var. *sigüín*	Tequila, Jal.	Y/S
A. tequilana var. *bermejo*	Tequila, Jal.	Y/S
A. rhodacantha	Tepic, Nay.	S/D
A. rhodacantha	San Gabriel, Jal.	D/D
A. rhodacantha var. *ixtlero amarillo*	Zapotitlan de Vadillo, Jal.	Y/Y
A. rhodacantha	Canoas, Jal.	Y/Y
A. rhodacantha	Pueblitos, Jal.	Y/D
A. rhodacantha	Alamos, Son.	D/D
A. rhodacantha	University City UNAM, CdMx	D/D
A. rhodacantha	Ejutla, Oax.	Y/D
A. rhodacantha	Sierra Barobampo, Sin.	S/D
A. angustifolia	Sayula, Jal.	Y/S
A. angustifolia	San Gabriel, Jal. (near Comala vista point)	Y/Y
A. angustifolia	Santiago de Pinos, Jal.	Y/Y
A. angustigolia	Huajuapan, Oax.	Y/D
A. angustifolia	Ahuacuotzingo, Gro.	S/D
A. angustifolia	Mazatlán, Sin.	Y/Y
A. angustifolia	Salvador Alvarado, Sin	D/D
A. angustifolia	Alamos, Son	Y/Y
A.angustifolia ssp. *rubescens*	Milpillas, Gro.	S/S
A. tequilana var. *espadín*	San Dionisio Ocotepec, Oax.	D/D
A. guadalajarana	Pueblitos, Jal.	D/D
A. guiengola	Santo Domingo Tehuantepec, Oax.	Y/Y
A. vilmoriniana	Batopilas, Chih.	D/D
A. isthmensis	Ocozocuautla, Chis.	Y/D
A. horrida	Taxco de Alarcón, Gro.	Y/Y
A. americana var. *oaxaquensis*	Tlacolula, Oax.	Y/D
A. parryi	La Palmita, Dgo.	D/D
A. zebra	Caborca Son.	D/D
A. aktites	Yavaros, Son	Y/Y
B. calcicola	Teposcolula, Oaxaca	Y/S

Table S6. List of Genbank accession numbers for *Mayahuelin* genes from the Agavoideae specimens studied in this work.

**Taxa**	**Location or origin**	**Specimen collection number**	**Genbank accession number**
A. tequilana var. *azul*	Tequila, Jal.	N.A.	MN913554
A. tequilana var. *mano larga*	Tequila, Jal.	JNS24	MN937354
A. tequilana var. *manso*	Tequila, Jal.	JNS27	MN937355
A. tequilana var. *sigüín*	Tequila, Jal.	JNS28	MN937356
A. tequilana var. *bermejo*	Tequila, Jal.	JNS29	MN937357
A. tequilana var. *espadín*	San Dionisio Ocotepec, Oax.	JNS63	MN937358
A. rhodacantha	Tepic, Nay.	JNS11	MN937359
A. rhodacantha	San Gabriel, Jal.	JNS40	MN937360
A. rhodacantha var. *ixtlero amarillo*	Zapotitlán de Vadillo, Jal.	JNS46	MN937361
A. rhodacantha	Canoas, Jal.	JNS48	MN937362
A. rhodacantha	Pueblitos, Jal.	JNS54	MN937363
A. rhodacantha	Alamos, Son.	AG9173	MN937364
A. rhodacantha	UNAM campus, CdMx	JE06	MN937365
A. rhodacantha	Ejutla, Oax.	AG10959	MN937366
A. rhodacantha	Sierra Barobampo, Sin.	DS1686	MT113953
A. angustifolia	Sayula, Jal.	JNS37	MN937367
A. angustifolia	San Gabriel, Jal.	JNS43	MN937368
A. angustifolia	Santiago de Pinos, Jal.	JNS05	MN946507
A. angustifolia	Huajuapan, Oax.	MRL87	MN946508
A. angustifolia	Ahuacuotzingo, Gro.	ES01	MN946509
A. angustifolia	Mazatlán, Sin.	DS1663	MT113954
A. angustifolia	Salvador Alvarado, Sin.	DS1664	MT113955
A. angustifolia	Alamos, Sonora	DS1682	MT113956
A. angustifolia ssp. *rubescens*	Milpillas, Gro.	SC04	MN946510
A. americana var. *oaxacensis*	Tlacolula, Oax.	AG10531	MN946511
A. guadalajarana	Pueblitos, Jal.	JNS58	MN946512
A. guiengola	Santo Domingo Tehuantepec, Oax.	AG5685	MN946513
A. horrida	Taxco de Alarcón Gro.	SC06	MN946514
A. isthmensis	Ocozocuautla, Chis.	AG4177	MN946515
A. parryi	La Palmita, Durango	AG4968	MN946516
A. vilmoriniana	Batopilas, Chih.	6000	MN946517
A. zebra	Caborca, Son.	AG8876	MN946518
A. aktites	Yavaros, Son	DS1679	MT113957
Beschorneria calcicola	Teposcolula, Oax.	AG8771	MN946519

Table S7. Estimates of reconstruction implemented by Bayesian inference analyses using the parameters described under "Phylogenetic Analyses" of Materials and Methods section. Abreviations: ESS, estimated sample size; PRSF, potential scale reduction factor; TL, tree length; r, reversible substitution rate between the indicated bases; pi, stationary frequency of indicated codon; omega, Nonsynonymous/synonymous rate ratio.

Parameter	Mean	Variance	Lower	Upper	Median	total ESS	PSRF
TL	0.23717	0.00038	0.20176	0.27790	0.23657	4511	1.00106
r(A<->C)	0.22425	0.00115	0.15769	0.28965	0.22335	2379	0.99979
r(A<->G)	0.26736	0.00127	0.20164	0.33773	0.26619	1810	1.00182
r(A<->T)	0.08651	0.00055	0.04246	0.13191	0.08433	2588	0.99975
r(C<->G)	0.12378	0.00074	0.07187	0.17619	0.12161	2573	1.00048
r(C<->T)	0.15964	0.00122	0.09647	0.23001	0.15769	1760	0.99977
r(G<->T)	0.13846	0.00100	0.07639	0.19743	0.13677	2630	1.00053
omega	1.91398	0.24412	1.10146	2.91453	1.85211	3438	1.00065
pi(AAA)	0.02561	0.00005	0.01289	0.03961	0.02490	452	1.00074
pi(AAC)	0.04031	0.00008	0.02341	0.05889	0.03988	444	1.00232
pi(AAG)	0.02147	0.00004	0.01036	0.03427	0.02082	461	1.00062
pi(AAT)	0.02812	0.00007	0.01388	0.04512	0.02720	150	1.00212
pi(ACA)	0.03086	0.00007	0.01675	0.04713	0.03025	309	1.00160
pi(ACC)	0.02199	0.00005	0.01013	0.03643	0.02135	593	0.99981
pi(ACG)	0.02121	0.00006	0.00835	0.03682	0.02024	327	1.00168
pi(ACT)	0.01018	0.00002	0.00199	0.02015	0.00927	408	1.00225
pi(AGA)	0.01720	0.00004	0.00658	0.02977	0.01628	233	1.00284
pi(AGC)	0.00406	0.00001	0.00005	0.00966	0.00345	591	0.99982
pi(AGG)	0.02493	0.00006	0.01113	0.04106	0.02400	479	1.00164
pi(AGT)	0.00549	0.00002	0.00016	0.01283	0.00464	421	1.00523
pi(ATA)	0.02979	0.00008	0.01389	0.04749	0.02904	340	1.00245
pi(ATC)	0.01491	0.00003	0.00438	0.02580	0.01398	467	0.99979
pi(ATG)	0.01562	0.00004	0.00483	0.02766	0.01485	568	1.01062
pi(ATT)	0.01085	0.00004	0.00074	0.02333	0.00962	258	0.99976
pi(CAA)	0.01512	0.00004	0.00470	0.02687	0.01429	400	1.00006
pi(CAC)	0.01128	0.00002	0.00273	0.02045	0.01057	441	1.00158
pi(CAG)	0.02663	0.00008	0.01151	0.04493	0.02552	480	1.00196
pi(CAT)	0.01110	0.00003	0.00189	0.02143	0.01022	325	1.00282
pi(CCA)	0.00802	0.00002	0.00076	0.01735	0.00712	506	1.00359
pi(CCC)	0.01030	0.00003	0.00223	0.02121	0.00939	408	1.00441
pi(CCG)	0.00803	0.00002	0.00093	0.01653	0.00725	465	1.00417
pi(CCT)	0.01397	0.00004	0.00269	0.02582	0.01320	338	0.99986
pi(CGA)	0.00540	0.00001	0.00010	0.01312	0.00459	363	0.99994
pi(CGC)	0.01481	0.00004	0.00360	0.02677	0.01412	522	0.99987

pi(CGG)	0.00251	0.00001	0.00001	0.00762	0.00176	567	1.00635
pi(CGT)	0.00655	0.00002	0.00015	0.01527	0.00550	543	1.00609
pi(CTA)	0.00738	0.00002	0.00083	0.01625	0.00658	381	0.99980
pi(CTC)	0.02260	0.00005	0.00907	0.03637	0.02186	348	1.00370
pi(CTG)	0.02591	0.00007	0.01169	0.04421	0.02474	527	1.00064
pi(CTT)	0.00623	0.00002	0.00036	0.01478	0.00531	492	1.00075
pi(GAA)	0.01884	0.00004	0.00729	0.03090	0.01830	499	1.00413
pi(GAC)	0.03983	0.00007	0.02356	0.05554	0.03926	632	1.00105
pi(GAG)	0.02354	0.00005	0.01151	0.03775	0.02270	434	1.00194
pi(GAT)	0.01437	0.00003	0.00513	0.02519	0.01343	341	0.99988
pi(GCA)	0.04081	0.00009	0.02311	0.05931	0.04004	405	1.00159
pi(GCC)	0.01672	0.00004	0.00525	0.02889	0.01572	469	0.99981
pi(GCG)	0.01884	0.00004	0.00793	0.03228	0.01789	539	1.00478
pi(GCT)	0.01323	0.00003	0.00385	0.02583	0.01250	493	1.00328
pi(GGA)	0.01741	0.00004	0.00676	0.02951	0.01692	536	1.00024
pi(GGC)	0.02808	0.00006	0.01260	0.04390	0.02733	416	1.00012
pi(GGG)	0.01453	0.00003	0.00485	0.02573	0.01387	360	1.00012
pi(GGT)	0.01790	0.00005	0.00635	0.03237	0.01713	376	1.00722
pi(GTA)	0.01081	0.00002	0.00233	0.01979	0.01035	375	1.00687
pi(GTC)	0.01044	0.00002	0.00297	0.02027	0.00976	470	1.00493
pi(GTG)	0.02594	0.00006	0.01290	0.04276	0.02490	346	1.00882
pi(GTT)	0.00555	0.00002	0.00017	0.01371	0.00448	462	1.00002
pi(TAC)	0.02868	0.00007	0.01415	0.04517	0.02793	341	1.00223
pi(TAT)	0.00863	0.00003	0.00120	0.01885	0.00753	363	0.99977
pi(TCA)	0.00569	0.00002	0.00007	0.01364	0.00483	309	1.00403
pi(TCC)	0.01197	0.00003	0.00295	0.02199	0.01111	450	0.99982
pi(TCG)	0.00264	0.00001	0.00001	0.00799	0.00189	371	1.00054
pi(TCT)	0.00897	0.00002	0.00104	0.01858	0.00819	360	1.00132
pi(TGC)	0.00724	0.00002	0.00091	0.01591	0.00643	352	1.00680
pi(TGG)	0.01807	0.00005	0.00595	0.03208	0.01727	350	1.00676
pi(TGT)	0.00292	0.00001	0.00000	0.00885	0.00201	325	1.00009
pi(TTA)	0.00252	0.00001	0.00000	0.00735	0.00173	646	0.99979
pi(TTC)	0.03789	0.00009	0.01977	0.05573	0.03711	239	0.99985
pi(TTG)	0.01546	0.00004	0.00400	0.02779	0.01457	379	1.00321
pi(TTT)	0.01404	0.00004	0.00367	0.02554	0.01339	428	1.00046
